# Radiation Detectors and Sensors in Medical Imaging

**DOI:** 10.3390/s24196251

**Published:** 2024-09-26

**Authors:** Christos Michail, Panagiotis Liaparinos, Nektarios Kalyvas, Ioannis Kandarakis, George Fountos, Ioannis Valais

**Affiliations:** Radiation Physics, Materials Technology and Biomedical Imaging Laboratory, Department of Biomedical Engineering, University of West Attica, Ag. Spyridonos, 12210 Athens, Greece; cmichail@uniwa.gr (C.M.); liapkin@uniwa.gr (P.L.); nkalyvas@uniwa.gr (N.K.); gfoun@uniwa.gr (G.F.); valais@uniwa.gr (I.V.)

**Keywords:** radiation detectors, optical sensors, medical imaging systems, diagnostic radiology, nuclear medicine, artificial intelligence, deep learning

## Abstract

Medical imaging instrumentation design and construction is based on radiation sources and radiation detectors/sensors. This review focuses on the detectors and sensors of medical imaging systems. These systems are subdivided into various categories depending on their structure, the type of radiation they capture, how the radiation is measured, how the images are formed, and the medical goals they serve. Related to medical goals, detectors fall into two major areas: (i) anatomical imaging, which mainly concerns the techniques of diagnostic radiology, and (ii) functional-molecular imaging, which mainly concerns nuclear medicine. An important parameter in the evaluation of the detectors is the combination of the quality of the diagnostic result they offer and the burden of the patient with radiation dose. The latter has to be minimized; thus, the input signal (radiation photon flux) must be kept at low levels. For this reason, the detective quantum efficiency (DQE), expressing signal-to-noise ratio transfer through an imaging system, is of primary importance. In diagnostic radiology, image quality is better than in nuclear medicine; however, in most cases, the dose is higher. On the other hand, nuclear medicine focuses on the detection of functional findings and not on the accurate spatial determination of anatomical data. Detectors are integrated into projection or tomographic imaging systems and are based on the use of scintillators with optical sensors, photoconductors, or semiconductors. Analysis and modeling of such systems can be performed employing theoretical models developed in the framework of cascaded linear systems analysis (LCSA), as well as within the signal detection theory (SDT) and information theory.

## 1. Introduction-Background

Medical imaging instrumentation consists of radiation sources and radiation detectors built around the human body or some other object of biomedical interest. This radiation can be either indirectly ionizing (i.e., X-rays or gamma-rays) or non-ionizing (magnetic or ultrasonic pulses in the radiofrequency range, optical imaging). Detection of ionizing radiation is based on X-ray or γ-ray energy deposition through photon-matter interaction effects (photoelectric, Compton, annihilation) [1,2,3,4,5,6,7,8,9,10,11,12,13,14,15,16,17,18,19].

On the basis of their measurement principle, detectors are divided into two main categories:

(i) *Energy integrating devices (EID)* producing an output signal directly proportional to the total radiation energy absorbed within the detector mass. In planar-projection imaging (see below), these detectors perform a direct mapping of x and γ-ray photon interaction points (mostly used in diagnostic radiology and in portal imaging, i.e., imaging in radiation therapy with linear accelerators). Photons that deposit different amounts of energy in the detector or are absorbed at different depths and create unequal output pulses produce signals of different intensity. This causes statistical variations in the signal intensity per measured photon, which constitutes noise (Lubberts and Swank effects) [1,5,6,7,10,12,13,14,15,16,18,19].
(ii) *Photon counting devices (PCD)* based on spectrometric recording of the number of photons and on calculation of coordinates of the radiation interaction points with the detector [used in nuclear medicine]. Photon counting produces a series of pulses, one by one, corresponding to the measured incident X-ray or γ-ray photons. Of central importance for the spectrometric operation of photon counting systems is the *pulse height analyzer*. Through this device, the useful part of the incident radiation energy spectrum is selected in order to assure detection of primary radiation (e.g., by rejecting scattering photons or photons from other sources). This selection can be achieved by the *energy window.* Photons are first converted into electronic pulses and, if they can pass through this window, are recorded and assumed to be of identical pulse amplitude. Thus, no statistical differentiation appears. PCDs are also used in some diagnostic radiology systems, e.g., digital mammography and computed tomography [2,4,8,9,11,14,17,18].

Based on the nature of the diagnostic information they provide, imaging systems and methods are divided into: (i) *morphological imaging,* depicting shape, dimensions, and coordinates of anatomical structures as well as mechanical movement or flow of biological fluids, i.e., in cardiovascular mechanisms, etc.; and (ii) *functional imaging,* seeking to detect mechanisms of biological-physiological nature, including processes at the molecular level. Morphological imaging is employed in diagnostic radiology, including ultrasound and magnetic resonance imaging as well as in portal imaging. Functional imaging is used in nuclear medicine, which is based on radiopharmaceuticals (*radioactive tracers*) administered to patients and then on recording of their behavior inside the patient’s body by detecting γ radiation [1,2,3,4,5,6,7,8,9,10,11,12,13,14,15,16,17,18].

Image creation procedures are divided into three basic stages: (i) radiation detection; (ii) initial image formation (mapping radiation distribution in space—projective, tomographic reconstruction); and (iii) image processing (digital techniques, digital filters, etc.). Considering the techniques of image production, systems and methods are classified in two categories: (i) *projection imaging*, where three-dimensional objects (i.e., anatomical structures) are projected onto a two-dimensional area of the detector; and (ii) *tomographic imaging*, reconstructing cross-sectional images by employing mathematical algorithms and using measured data obtained through measurements on a three-dimensional object [14,15,16,17,18]. Tomographic images are created from projection data acquired at many different angles around the patient. Reconstruction techniques are classified in two major categories, i.e., *analytical* and *iterative techniques* (IR). *Filtered back projection* (FPB) (filtering of data before back projection) is the most popular analytical technique. In IR, images are reconstructed by iteratively optimizing a particular function. Along with the development of iterative algorithms, reconstruction techniques based on *deep learning algorithms* (DLR) have come into use [20,21,22,23,24,25,26,27,28,29,30].

Considering the signal generation techniques, imaging methods follow: (i) *Transmission techniques*, where the radiation (e.g., from an X-ray tube, radioactive source, etc.) is produced outside the patient’s body and passes through it to hit the detector (case of diagnostic radiology); (ii) *emission techniques,* where the radiation source (e.g., in the form of a radioactive isotope) is administered inside the human body (in some anatomical area) and participates in the body’s functions. Then the radiation (radioactivity) is emitted through the human body, and after exiting, it hits the detector (nuclear medicine); (iii) *transmitter-receiver (pulse-echo) techniques,* where the source of the radiation (usually an ultrasonic pulse or a magnetic pulse) is emitted by a transmitter outside the human body and hits it, interacts with it, and then returns to the transmitter (through reflection, backscatter, electromagnetic induction) (ultrasound, magnetic resonance imaging) [14,15,16,17,18].

Most medical imaging radiation detectors incorporate scintillators/phosphors coupled to optical sensors, i.e., (i) large area hydrogenated amorphous silicon (a-Si:H) active matrix photodiode arrays (AMFPI), (ii) complementary metal oxide semiconductors (CMOS), (iii) charge-coupled devices (CCD) (for X-ray imaging), and (iv) photomultipliers (PMT), (v) avalanche photodiodes (APD), (vi) silicon multipliers (SiPM) (for detection of radionuclides). These detectors are often referred to as *indirect conversion or indirect detection* imaging systems. On the other hand, detectors based on photoconductors (e.g., amorphous selenium) or semiconductors (e.g., cadmium telluride) are characterized as *direct detection* systems. Indirect conversion systems may be considered as two-stage converters, i.e., one stage to convert ionizing radiation into light and a second stage to convert light into an electronic signal. Direct conversion refers to systems converting incoming radiation directly into an electronic signal. The most popular materials for such conversion are the amorphous selenium (a-Se) photoconductors and cadmium zinc telluride (CZT) semiconductors [1,3,12,13,19,31,32,33]. Si-based detectors have also been used in some systems [33]. In all cases, detecting materials and optical sensors are coupled to processing electronics, which constitute a necessary component of an integrated detector system.

Medical imaging systems can also be categorized according to their particular medical use, i.e., those belonging to *diagnostic radiology* (anatomic—morphological X-ray images) and those belonging to *nuclear medicine* (functional information using radioactivity). Portal imaging systems, used with linear accelerators of *radiation therapy,* are practically identical to those of diagnostic radiology, except that they detect higher energy photons in the range of several MeVs. The basic equipment included in the field of diagnostic radiology is: X-ray screen-film radiography (SFR), digital radiography (DR) and fluoroscopy (DF), full-field digital mammography (FFDM), computed radiography (CR), as well as X-ray computed tomography (CT), dental radiography, etc. Nuclear medicine equipment includes gamma camera-single photon emission computed tomography (SPECT) and positron emission tomography (PET) [1,2,3,4,5,6,7,8,9,10,11,12,13,14,15,16,17,18,19,31]. Finally, it is important to mention *phase-contrast X-ray imaging* (PCI), a morphological imaging based on the wave properties of photons that includes various imaging methods to depict information originating from changes in the phase of an X-ray beam penetrating an object [34]. In many cases, PCI ameliorates image contrast. Techniques related to phase information are also employed in MRI.

Regarding the market shares of medical imaging systems, the largest global market share (more than 35%) is held by projection diagnostic radiology and especially projection imaging systems (radiography, fluoroscopy, mammography, interventional radiology, dental radiography). X-ray computed tomography systems also have a large share of the global market. Overall, diagnostic radiology (both projection and tomographic) clearly accounts for more than 50% of the global medical imaging market shares [35]. For nuclear medicine, the market share is well below 10%. For the period 2017–2024, medical imaging technology is third, among fifteen medical technology sectors, in global market shares. However, it is worth noting that this share does not include the part of imaging technology that is incorporated in other technology areas, e.g., cardiology, dentistry, orthopedics, etc. [36].

**Figure 1:**
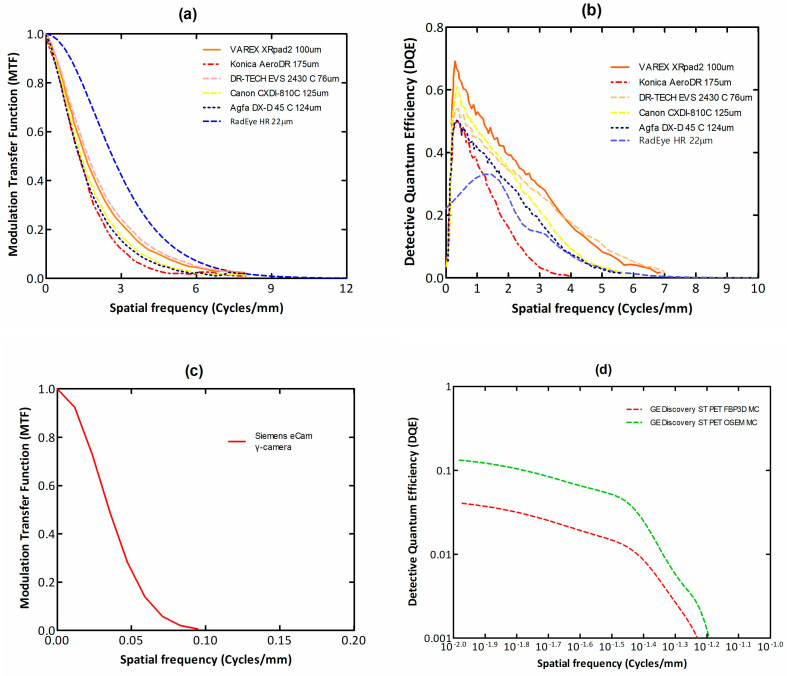
MTF (**a**,**c**) and DQE (**b**,**d**) data for X-ray and nuclear imaging systems. It can be seen that both MTF and DQE curves of the X-ray systems are higher and broader than the ones corresponding to nuclear medicine [40,41,45,46,47,48].

Finally summarizing, we could distinguish the main differences between the systems of diagnostic radiology (including radiation therapy) and those of nuclear medicine, as follows:

(i) Detectors used in diagnostic radiology are mostly energy-integrating. Exceptions are the systems of spectral CT and some digital mammography systems (see Section 5.1.4 and Section 5.2). In nuclear medicine, systems are photon counting, based on γ-ray spectrometry techniques, measuring photons one by one and producing corresponding electronic pulses.
(ii) In terms of the physical phenomena involved in the image formation process. In X-ray anatomical imaging, images are created after interaction of X-rays, first with human biological tissues and then with detector materials, through which they pass. In functional nuclear imaging, radionuclides (in the form of *radiopharmaceuticals*) are administered into the human body to track biological processes. γ-rays must not interact with the biological tissues, so that the emission from the anatomical structures reaches the detectors unaffected and interacts only with the detectors. Since always some interaction occurs, methods to correct for attenuation effects have been developed, some of them based on *artificial intelligence* techniques [4,5,14,15,37,38].
(iii) In the number of radiation photons used to create an image, e.g., in diagnostic radiology for a chest radiography (110 kVp, 3 mAs), this number is in the range of 15–16 × 10^6^ photons per mm^2^, while in nuclear medicine for a lung scan, this number is more or less 30–40 photons per mm^2^ and per second [4,5,39]. This difference has a decisive effect on the objective physical quality of the obtained image.
(iv) Energy and spectral distribution of radiation photons. In X-ray imaging, a spectrum of X-rays (filtered by Al, Mo, etc. filters, as well as by the human body) is employed with maximum energy ranging from 20 to 150 keV (mean energy is significantly lower). This spectrum is electronically produced by accelerating electrons in X-ray tubes [5,14,15]. In nuclear medicine, monochromatic γ-ray photons are used with energy depending on the radionuclide used, ranging from 140 keV (Tc 99 m) to 512 keV (annihilation photons). Radionuclides used in SPECT are mainly produced in nuclear reactors, and in most cases, delivered through radioactive generators (i.e., in the case of Tc-99 m) [4,17,18]. For PET, radionuclides are produced by cyclotrons. In portal imaging (in radiation therapy), the X-ray photon energy is spectrally distributed in the range of some MeVs, produced by linear accelerators. Monochromatic X-rays can also be produced in synchrotron accelerators (synchrotron radiation), in crystal/multilayer monochromator systems coupled to standard X-ray tubes, in X-ray fluorescence effects, and laser-driven technology [4,5,14,15,17].
(v) Objective image quality is also significantly different. For example, the spatial resolution (in line pairs per mm-lp/mm) in X-ray projection imaging is of the order of 3–10 lp/mm, depending on the modality. In ordinary X-ray CT, values can be higher than 1.5–1.9 lp/mm, while values better than 4 lp/mm have been achieved in one case. In nuclear medicine (e.g., gamma cameras-SPECT), spatial resolution is of the order of 0.07–0.10 lp/mm. Considering the PSF, i.e., image of a point source, the performance of ordinary CT is of the order of 0.5 mm (i.e., the full width at half maximum (FWHM) of the one-dimensional PSF curve) and 0.14 mm in some newer systems. For SPECT it may be of the order of several mm (i.e., 5–6 mm to more than 10 mm), and in PET it may be 4–5 mm or even lower, probably 2 mm. Making a comparison with extreme values, we mention X-ray mammography, in which the spatial resolution can reach a few tens of μm. However, the principal aim of nuclear medicine is the investigation of functional characteristics and not the accurate description of anatomy. The following Figure 1 shows a comparison of data on *modulation transfer function* (MTF), expressing image contrast and spatial resolution in the spatial frequency domain, and on *detective quantum efficiency* (DQE), expressing signal-to-noise ratio transfer in imaging systems. As it can be seen, by observing the roll-off of the MTF and the DQE curves, the image quality is significantly better in X-ray imaging (Figure 1). High frequencies correspond to small dimensions (spatial resolution is often estimated as the spatial frequency corresponding to MTF = 0.05). The curves corresponding to diagnostic radiology are higher and cover a broader range of frequencies and therefore yield more morphological diagnostic information throughout the range of object dimensions and especially in the small dimensions [40,41,42].
(vi) A very important aspect of detector operation is the speed of response of the entire detector system (*timing performance*). In nuclear medicine and especially in PET systems, particularly short response times are required. Parameters such as *coincidence resolution time* (CRT) and *single photon time resolution* (SPTR) have been defined to express the optical sensor temporal performance (SiPM) of such systems. SPRT values as low as some tens of ps have been reported, while 10 ps is a desired performance [43]. CRT values of some hundreds (214–325 ps) have been achieved. Although in projection X-ray imaging the requirements are less, the current development of computed tomography systems with photon counting detectors advocates designing systems with very fast response (tens of ms) [44].

Although not strictly related to detectors and sensors, a very important innovation in the field of medical imaging is the introduction and use of *artificial intelligence* (AI). Subfields such as deep learning, subfields of *machine learning* (ML), branching into *artificial neural networks* (ANN), *convolutional neural networks* (CNN), and *generative adversarial networks* (GAN) are successfully introduced in various fields of imaging applications. Deep learning algorithms are widely employed for image analysis in all imaging modalities, including segmentation, disease prediction, and tumor detection in DR, FFDM, CT, MRI, US, SPECT, and PET. Computer-aided diagnosis (CAD) systems using artificial intelligence algorithms are developed. In CT, AI based deep learning image reconstruction as well as post-processing techniques can improve image quality while keeping dose at low levels. In nuclear medicine, particularly, AΙ is employed in data correction (attenuation, scatter events) and determination of position of photon incidence. AI algorithms can also be used to improve image quality parameters, thus improving the performance of radiation detectors [20,21,23,24,25,26,27,28,29,30,37,38,49,50,51,52,53,54,55,56].

This review mainly concerns medical imaging systems based on ionizing radiation and corresponding systems of a hybrid nature. It should be noted, however, that very interesting groups of imaging systems use non-ionizing radiation, such as ultrasound, optical imaging (e.g., optical coherence tomography—OCT), and thermal imaging (near infrared imaging—NIR). An interesting presentation of these systems is given in the following publications [14,18,57]. Table 1 is a list of the commonly used medical imaging systems.

## 2. Theoretical Modeling-Signal and Noise

Analysis of the imaging systems is performed in both spatial and spatial frequency domains, where signals are decomposed in their spectral components [58,59,60,61,62,63,64,65,66,67,68,69,70,71,72,73,74,75,76,77,78,79,80,81,82,83]. The image formation process is generally described by an equation of the following form (Equation (1)):

(1) $$i\left(x,y\right)=\iint_{x,y}h\left(x-x^{\prime },y-y^{\prime }\right)f\left(x^{\prime },y^{\prime }\right)dxdy+n\left(x,y\right)=h\left(x,y\right)⊗f\left(x,y\right)+n\left(x,y\right)$$

*i*(*x*,*y*) represents the final image, *f*(*x*,*y*) is the unknown object to be imaged, and *h*(*x*,*y*) is the system’s *transfer function*, expressing the effect of the imaging system on the unknown object. The function *n*(*x*,*y*) expresses the noise (see next paragraphs), which is additive. If a Fourier transform is applied to the above equation, then the functions (now expressed with capital symbols) are transferred to the spatial frequency domain (*u*,*v*), which is often used to analyze systems’ performance. The convolution operation turns into a simple product (Equation (2)):

(2) $$I\left(u,v\right)=F\left(u,v\right)H\left(u,v\right)+N\left(u,v\right)$$

Medical imaging systems are generally considered to be approximately *linear* (see above equation) or linearizable and *stationary*. An imaging system is stationary if the functions expressing signal and noise transfer do not depend on the specific point of their measurement. A random process, such as the fluctuations in the number of photons that generate the statistical noise, is statistically stationary if the autocorrelation function is independent of the area of the image in which the measurement is made. Stationary property holds for both space and time; however, this is not always strictly met. In particular, digital imaging systems with images in the form of an array of pixels are characterized as *wide-sense cyclostationary systems* or *weak sense cyclostationary systems* (WSC) (i.e., periodically varying statistical properties, including second-order statistics such as the autocorrelation function). Another property of random processes is *ergodicity*. Ergodicity means that the average value of measurements at various points in the image is equal to the average value of repeated measurements at the same point. If the input function *f*(*x*,*y*) is represented by an *impulse (Dirac delta function)*, the transfer function *h* is often defined as the PSF of the detector.

A property characterizing the image formation process is *sampling.* Digital images consist of a large number of sampling points in space. Each point corresponds to an image value expressing the integral of image values at points in a small area around this point. This small area is defined by the *aperture* of the digital detector element (del). The distance among sampling points defines the *sampling pitch*. Sampling is analytically expressed as the product of *f*(*x*,*y*) with a comb function (infinite sum of Dirac delta functions centered at the sampling points, Equation (3)):

(3) $$f_{s}\left(x,y\right)=f(x,y)\sum_{nx=1}^{\infty }\sum_{ny=1}^{\infty }\delta (x-n_{x}a,{y-n}_{y}a)$$

where *f_S_* is the sampled *f*(*x*,*y*), *a* is the spacing between the delta functions, and *n_x_*, *n_y_* are integers. Related to the sampling pitch is the *fill factor*, defined as the ratio of the detector element’s (pixel) area over the area defined by the actual detector pitch [58]. If sampling, particularly in a digital system, is not acceptably fine (broad aperture), *aliasing* may occur, i.e., higher frequencies appear as lower. To avoid aliasing, the *Nyquist-Shannon sampling theorem* must be fulfilled (i.e., sampling frequency at least twice the signal bandwidth).

*Noise* (i.e., random spatial variations of the number of detected radiation photons) is important in medical imaging methods employing ionizing radiations. Noise originates from random signal generation and is related to the number of radiation photons (radiation dose). Medical imaging systems detect signals in a noisy background and are referred to as *quantum-limited systems.* This holds because in such methods care must be taken to reduce patient radiation burden by decreasing incident radiation, similarly affecting input signal level. Thus, considering the physical and technical requirements in designing and evaluating imaging systems, it should be taken into account that a human observer detects signals in a noisy background (i.e., the ability to detect an object depends on the signal-to-noise ratio (SNR)). Hence, the performance and reliability of imaging systems are assessed by the signal-to-noise ratio, which should be the highest possible. SNR is often assessed by the detective quantum efficiency defined as (Equation (4)):

(4) $$DQE={SNR}_{out}^{2}/{SNR}_{in}^{2}$$

where *SNR_out_* and *SNR_in_* denote the signal-to-noise ratio at the output and input of an imaging system, respectively. Within this context, the following definitions can be given: (i) input signal: flux of incident radiation photons; (ii) output signal: emitted light flux or generated electrical charge at the output of the detector; (iii) input noise: statistical variance in the spatial distribution of incident photons, i.e., the so-called *primary quantum noise*; (iv) output noise: statistical variance in the spatial distribution of emitted light photons or produced electrons at the detector output. The so-called *secondary quantum noise* is related to the creation and propagation of secondary carriers (light photons, electrons). DQE may be expressed through MTF, defined as the spatial frequency-dependent modulation transfer function, normalized to unity (or the modulation contrast of the image over the modulation contrast in the object) [58] and the *noise power spectrum* (NPS), expressing noise in the spatial frequency domain. If the properties at one image pixel do not depend on the properties of surrounding pixels, the noise is characterized as *uncorrelated noise.* However, since secondary carriers (light photons, electrons) spread out in a broader area, pixel values become *correlated*. DQE, MTF, and NPS are the principal descriptors of an imaging detector response in the spatial frequency domain. MTF is related to PSF since it can be estimated as the Fourier transform of the *line spread function* (LSF), which in turn is estimated as the integral of the two-dimensional PSF across a direction, e.g., direction y.

**Figure 2:**
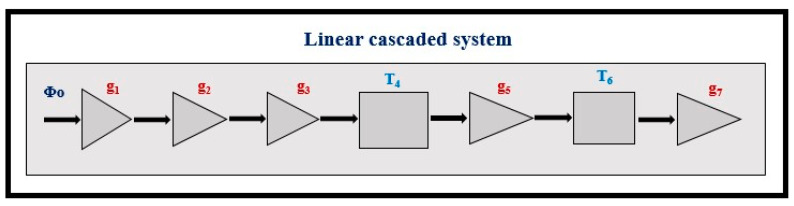
Schematic representation of an example of a linear cascaded system corresponding to an indirect conversion detector, Φ_0_: input signal (radiation photon flux), g_1_: quantum detection efficiency, g_2_: intrinsic conversion efficiency of radiation energy into light, g_3_: light transmission efficiency, T_4_: spreading stage of light within the scintillator material, g_5_: quantum gain of optical sensor (conversion of light into electrons), T_6_: spreading stage of electrons within optical sensor, g_7_: electronic processing.

The signal and noise propagation through an imaging system is described by the theory of *linear cascaded systems analysis* (LCSA)*,* where the imaging system is considered as a sequence of discrete cascaded stages, through which signal and noise are transferred (Figure 2). Details of the analysis that follows can be found in the following publications [59,64,66,67,69,70,71]. These stages are specific processes within the individual system parts, e.g., the stage of radiation detection, the stage of conversion into secondary carriers (light photons or electron—hole pairs), the propagation of secondary carriers inside the detector material, the absorption and conversion of the light into an electronic signal in the optical sensors, the digitization of the signal, etc. A key property of cascaded stages is that the output signal of one stage is the input to the next. Cascaded stages are of two types: (a) signal amplification or quantum gain stages and (b) signal spreading or blurring stages. Gain and spreading stages can be either stochastic stages or deterministic. In gain stages, changes in the number of carriers (quanta, electrons) may occur, e.g., in converting X-rays into optical photons, the number of quanta increases. In some cases, gain may be unity. In deterministic gain stages, the number of output quanta can be precisely determined from input quanta. In stochastic gain stages, the number of output quanta follows a certain statistical distribution, having a mean value and a statistical variance. Signal spreading stages are characterized by the spatial distribution of carriers (e.g., due to optical scattering in scintillators). The distribution of carriers in the detector’s output surface is described by the PSF (in space domain) and by the MTF (in frequency domain). One example of a deterministic stage is the input “aperture” of a photodiode or CCD cell in detectors (i.e., the optical photons are incident and distributed on the surface of the photodiode).

Each gain stage is characterized by: (a) an average gain, denoted by *g_i_*, and (b) an average gain variance, denoted by *σ_gi_*^2^. The input signal in a stage (either amplification or scattering) is described by a function *Φ_ι_*_−1_(*x*,*y*) expressing the distribution of quanta on the corresponding input (quantum flux or photon flux). The output signal is described by a function *Φ_ι_*(*x*,*y*). The input noise is expressed by the corresponding input NPS_i−1_. Accordingly, the output noise is described by the output noise power spectrum NPS*_i_*. In a gain stage, between input and output signal, the relationship applies (Equation (5)):

(5) $$\bar{\Phi_{i}}\left(x,y\right)={\bar{g}}_{i}{\bar{\Phi }}_{i-1}(x,y)$$

If it is a stochastic gain stage, the above quantities express average values of the input signal, gain, and output signal respectively. For stochastic and deterministic spreading stages, the output signal is written, respectively (Equation (6)):

(6) $$\bar{\Phi_{i}}\left(x,y\right)=\bar{\Phi_{i-1}}(x,y)**sP_{i}(x,y)\;\bar{\Phi_{i}}\left(x,y\right)=\bar{\Phi_{i-1}}(x,y)**\Pi_{i}(x,y)$$

where **_S_P(*x*,*y*) denotes the stochastic spreading process and is often referred to as stochastic convolution. The function *Pi*(*x*,*y*) is the PSF (random spread of quanta, e.g., optical photons in scintillators) [PSF can be defined as the response of a system to the Dirac delta function]. *Π_i_*(*x*,*y*) is the *rect*(*x*,*y*) function expressing the input aperture of the optical sensor, e.g., a CCD cell or a photodiode. From the properties of the binomial distribution, it follows that in the case of cascaded gain stages expressed by a binomial distribution, these stages can be replaced by a single total stage, also with a binomial distribution. Thus, the total average gain of several stages can be expressed as (Equation (7)):

(7) $$\bar{\Phi_{out}}\left(x,y\right)=\Pi_{i}\bar{g_{i}}\cdot \bar{\Phi_{0}}\left(x,y\right)=\bar{g_{Τ}}\bar{\Phi_{0}\left(x,y\right)}$$

For spreading (blurring) stages, expressed by PSF and MTF in the spatial frequency domain, it may be written (Equation (8)):

(8) $${MTF}_{out}\left(v\right)=\prod_{j}{MTF}_{j}(v)$$

Correspondingly, the total noise ($\sigma_{out}^{2}$) of the system has been expressed by the following relation (Equation (9)):

(9) $$\sigma_{out}^{2}=\bar{g_{Τ}^{2}}\sigma_{0}^{2}(x,y)+\sigma_{g}^{2}\bar{\Phi_{0}(x,y)}+\sum_{\iota }\sigma_{add}^{2}$$

$\sigma_{g}^{2}$ is the variance in the total gain, $\sigma_{0}^{2}$ is the input signal variance. The last term in the above equation corresponds to additive electronic noise. Details can be found in the above-referenced publications.

*Noise and signal-to-noise ratio*: radiation-based medical imaging systems are referred to as *quantum-limited systems.* A signal is detected within a noise background. Analysis of radiation detection processes assumes Poisson statistics for γ-ray or X-ray photons, for light photons, and binomial distribution probability for the radiation detection efficiency and for carrier (light photons, charges) propagation, e.g., LTE, etc. E.g., the variance in a binomial distribution may be written (Equation (10)):

(10) $$\sigma_{g_{i}}^{2}={\bar{g}}_{i}(1-{\bar{g}}_{i})$$

where ${\bar{g}}_{i}$ is the mean value of either quantum detection efficiency or/and light transmission efficiency. Under this assumption, the effect of noise in a three-stage indirect conversion detector has been expressed [60,63] as the variance in the number of light photons plus the additive noise of the electronic components of the imaging system, given as (Equation (11)):

(11) $$\sigma_{T}^{2}={\bar{\Phi }}_{g_{1}}{\left(g_{2}g_{3}\right)}^{2}+{\bar{\Phi }}_{g_{1}g_{2}g_{3}}+\sigma_{add}^{2}$$

The first term in the above equation expresses the uncorrelated noise component, while the second term gives the correlated noise. Since medical imaging detectors seek to determine signals in a noisy background, it is of primary importance to specify the signal-to-noise ratio. In the zero frequency case (space domain), this is often expressed through the *zero-frequency detective quantum efficiency*, given by the Equation (12) [1,3,19]:

(12) $$DQE=\eta_{Q}A_{s}=g_{1}\frac{m_{1}^{2}}{m_{0}m_{2}}$$

where *η_Q_* is the quantum detection efficiency (QDE), expressing the probability of interaction of a radiation photon in the detector, and *A_S_* is the so-called Swank factor related to the statistical fluctuations in the number of emitted light photons (or charge carriers in direct conversion detectors) per absorbed X-ray photon. $m_{i}$ are the zeroth, first, and second moments of the statistical distribution of the number of light photons (or charge carriers) emitted per X-ray absorbed. *m*_2_ increases with the width of the statistical distribution, while for a perfect detector $A_{s}=1$. Zero-frequency DQE, (i.e., DQE in the space domain) accounts for the fluctuation in the detector signal per incident photon, although these photons may be of equal energy detected. The effect of K-fluorescence (i.e., the emission of characteristic X-rays after photoelectric absorption, which may escape or be reabsorbed away from the point of their creation) and the Lubbert’s effect [80] (i.e., variations in the depth of absorption of incident photons create variations in signal intensity and spreading-PSF) are among the various noise sources affecting output noise. In the case of X-rays having a spectral distribution, the corresponding spectrum must be taken into account.

As it has been shown after the work of Rabbani, Van Meter, and Shaw [59,63,64,67], who used statistical moment-generating functions in the spatial frequency domain, the noise power spectrum, for the three kinds of stages. i.e., quantum gain, stochastic gain, and deterministic gain, respectively, have been written as follows [64,67,71] (Equations (13)–(15)):

(13) $${NPS}_{n}\left(u\right)={{\bar{g}}_{n}}^{2}{NPS}_{n-1}\left(u\right)+\sigma_{g_{n}}^{2}{\bar{\Phi }}_{n-1}$$

(14) $${NPS}_{n}\left(u\right)[{NPS}_{n-1}\left(u\right)-{\bar{\Phi }}_{n-1}]{MTF}_{n}^{2}(u)+{\bar{\Phi }}_{n-1}$$

(15) $${NPS}_{n}\left(u\right)={NPS}_{n-1}\left(u\right){MTF}_{n}^{2}(u)$$

Similarly, the spatial frequency-dependent DQE has been studied by Zweig [59,83,84], in the case of a system with cascaded gain stages characterized by a Poisson distribution and based on the noise transfer theory of Rabbani et al. [63] In the framework of this theory, in the Fourier-based approach, DQE has been expressed through Equation (16):

(16) $$DQE\left(u\right)=\frac{1}{1+\frac{1+\varepsilon_{g_{1}}{MTF}_{1}^{2}(u)}{{{\bar{g}}_{1}MTF}_{1}^{2}(u)}+\cdots +\frac{1+\varepsilon_{g_{N}}{MTF}_{N}^{2}(u)}{{{\bar{g}}_{1}\ldots {\bar{g}}_{N}MTF}_{1}^{2}\left(u\right)\ldots {MTF}_{N}^{2}\left(u\right)}}$$

*ε_g_*_1_ ($\varepsilon_{g_{1}}=\left(\frac{\sigma_{g_{n}}^{2}}{{\bar{g}}_{i}}\right)-1$) is a parameter expressing the degree of deviation from the Poisson distribution (of the statistical distribution of the quanta in stage (i)). Therefore, the DQE of an imaging system is affected by the values of the respective stage gain (*g_i_*) and the corresponding values of *MTF_I_*(*u*). The DQE analysis based on the above Zwieg relation is characterized as a “particle-based approach”. In the Poisson distribution, $\sigma_{g_{i}}^{2}={\bar{g}}_{i}$, so it will be ε_gi_ = 0. Through the above relationship, the effect of the MTF of each separate stage on the overall DQE of the system is clarified. Each stage characterized by spreading (of photons or electrons) has its own MTF, which degrades the overall DQE.

According to the above relationship, the increasing number of signal conversion stages decreases DQE due to the increased number of points of signal loss and noise introduction. For instance, in the case of the conversion of radiation energy into light, in scintillators, the number of photons increases, but the energy has large losses of the order 80–90%.

In *Signal detection theory*, the *signal difference to noise ratio* (SdNR) or *contrast to noise ratio* (CNR) has been defined as (Equation (17)):

(17) $$SdNR=\frac{(N_{B}-N_{o})A}{\sqrt{N_{B}A}}$$

where *N*_0_ is the signal in the area of the imaged object and *N_B_* is the background signal). *A* is the imaged surface of the object. The product *NA* expresses the number of photons. Within this theory, the *Rose model* has been developed, stating that in order for a human observer to detect an object, SdNR should be higher than a certain threshold, i.e., SdNR≥k, where *k* is approximately equal to 5 and depends on the human observer capabilities [64,77,78,81,82].

In the context of *information theory*, the concept of *information capacity* has been introduced by Shannon in order to estimate the amount of mathematical information in bits per unit area (Equation (18)) [85,86,87,88,89,90,91]:

(18) $$IC=\underset{T\to \infty }{\lim}\frac{{log}_{2}N_{S}}{T}=n_{p}{log}_{2}N_{S}$$

*N_S_* is the number of different signal intensities distinguished by a system, *T* is the duration of the signal, and *np* is the number of pixels per unit image area. It has been shown that for an imaging system, IC can be expressed as (Equation (19)):

(19) $$IC=\pi \int_{0}^{\infty }{log}_{2}\left[1+{SNR}_{0}^{2}\left(v\right)\right]vdv=\pi \int_{0}^{\infty }{log}_{2}\left[1+NEQ\left(v\right)\right]vdv$$

where NEQ is the noise equivalent quanta.

## 3. Materials for Radiation Converters

Regardless of the physical phenomenon on which the operation of a radiation detector is based, there are two basic properties that characterize the detecting materials [32,42,61,92,93]:

1. The efficiency of detecting (i.e., attenuating or absorbing) incoming radiation. Detection and absorption of radiation depend on interaction effects such as the photoelectric effect, the Compton effect, and pair production. Fast electrons liberated after these effects interact in the detector material, creating electron-hole pairs and then, after deexcitation, light photons [94,95]. This efficiency is often expressed as the gain g_1_ of the first stage in LCSA, i.e., a quantum gain stage associated with a binomial distribution.
2. The efficiency to convert the absorbed radiation into a measurable output signal (light or charge). This property is principally determined by the *forbidden energy gap* between the valence and the conduction energy bands of detector materials. It is often characterized as the g_2_ quantum gain stage.

The first property is expressed by QDE and by the energy absorption efficiency (EAE) [5,32,42]. EAE is the fraction of energy deposited at points of first interaction in the detector material. QDE is given as (Equation (20)):

(20) $$g_{1}\left(E\right)=\eta_{Q}=QDE\left(E\right)=(1-e^{-(\mu_{tot,t}(E)/\rho )w})$$

where $\mu_{tot,t}(E)/\rho$ is the total X-ray mass attenuation coefficient of the material. To determine EAE, the ratio of the mass energy absorption coefficient over the total mass attenuation coefficient is required (Equation (21)):

(21) $$g_{1}\left(E\right)=\eta_{E}=EAE\left(E\right)=(\frac{\mu_{tot,en}(E)}{\mu_{tot,t}(E)})(1-e^{-(\mu_{tot,t}(E)/\rho )w})$$

**Figure 3:**
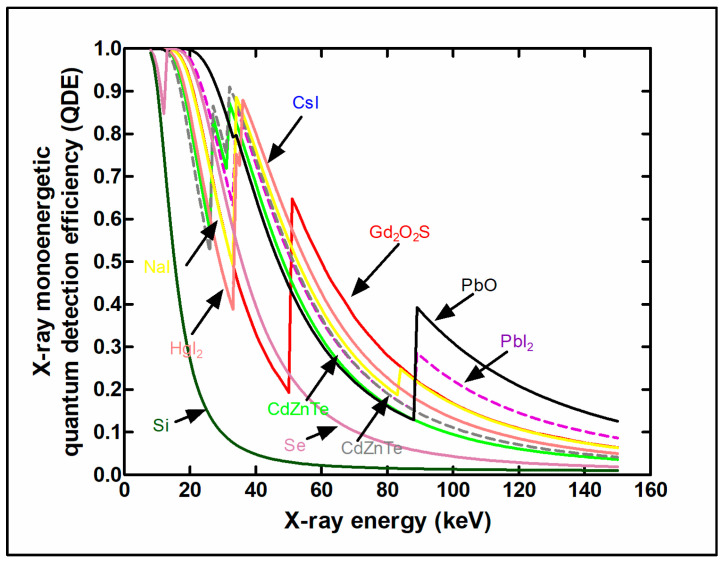
Comparison of QDE for various detector materials. The K-absorption edge, due to the photoelectric effect in the K shell of heavy elements, is clearly shown. CsI and Gd_2_O_2_S show high values in the X-ray imaging energy range, while Pb-based materials are more efficient at higher energies.

$\mu_{tot,en}(E)/\rho$ is the total mass energy absorption coefficient, and w denotes material thickness (surface density). The coefficient of energy absorption represents the average fraction of the kinetic energy of secondary charged particles that is locally deposited in the detector mass and excludes energy losses due to Bremsstrahlung radiation by electrons as well as K-fluorescence effects [5]. The coefficients of total attenuation and energy absorption depend strongly on (1) the effective atomic number of the material and (2) the energy of the K-edge of photoelectric absorption. A sudden increase in the values of these coefficients is observed once the incident photons exceed this energy. In the case of poly-energetic beams used in diagnostic radiology, QDE, and EAE are averaged over the X-ray spectrum. Both QDE and EAE depend on material thickness w and on photon energy *E*. A direct consequence of high QDE and EAE is the reduction of the dose to the patient for a given level of image quality. Another index indicating detection efficiency is the product, $\rho Z_{eff}^{k}$ (ρ: density, $Z_{eff}^{k}$ effective atomic number, and *k* depends on energy, being approximately 3) [96,97]. Figure 3 shows QDE variation with incident photon energy for various detector materials. The sudden increase due to the K-absorption edge is clearly shown for some materials.

A particular definition of detection efficiency ε_0_ has been given for detectors used in nuclear medicine as ε_0_ = ε_i_ε_g_, where ε_i_ is the *intrinsic efficiency*, equivalent to QDE, and ε_g_ is the *geometric efficiency* expressing the fraction of emitted γ-ray photons reaching the surface of the detector (depending on the solid angle) [4].

*Energy resolution* (ER), expressing the spectral discrimination of the energy of X-ray and γ-ray photons, is also related to the accuracy of radiation detection, which is required particularly in nuclear medicine applications. ER is defined as $R_{E}=E/\Delta E$, where *E* is the energy of radiation photons and Δ*E* is the full width at half maximum of the spectral line [4].

The whole process of radiation detection has some inherent drawbacks.

1. the range of the fast electrons liberated after the absorption of an incident photon may be long (e.g., 20–30 μm). Thus, a difference between the point of incident photon absorption and the point of secondary carrier (light photons, electrons) creation occurs. This affects the accuracy of projection and may degrade spatial resolution.
2. the K-fluorescence emission, e.g., X-ray photons emitted from the K-shell of the material’s heavy atom, after photoelectric absorption of an incident radiation photon. In planar projection detectors, these photons can be reabsorbed away from the point of initial photon incidence and degrade projection accuracy.
3. the oblique incidence of X-rays and γ-rays, if they are emitted by a point source, that creates blurring effects.
4. their absorption at varying depths within the mass of the scintillator results in slight variations in the signal intensity since, correspondingly, secondary carriers (light photons, electrons) travel different distances to exit detector material.

**Figure 4:**
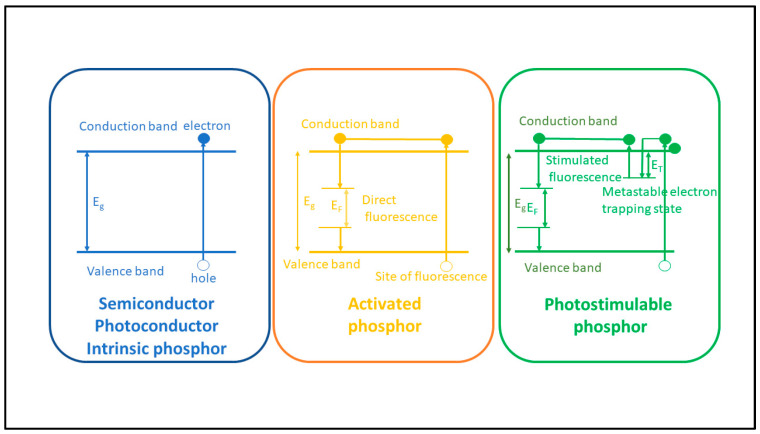
Energy level diagrams for semiconductors, scintillators (activated phosphor), and photostimulable phosphors. The forbidden energy band gap is shown.

Concerning the conversion efficiency of the absorbed radiation (energy or photons) into signal carriers (light photons, electron pairs), the role of the forbidden energy band gap (shown in Figure 4) must be noticed, which negatively affects this efficiency. As an example, we mention that the *intrinsic conversion efficiency* (ICE) of radiation energy into light in scintillators (*η_C_ ≈ hf/E_G_*) is of the order of 5–20%.

### 3.1. Luminescence-Scintillators and Phosphors

By definition, luminescence (fluorescence or phosphorescence) is the conversion of the absorbed radiation X, γ, etc., into light. Luminescent materials, in various forms, are most commonly used in medical imaging sensors [92,93,96,97,98,99,100,101,102,103,104,105,106,107,108]. They are available in a wide variety of compounds and forms, and research into them is extensive and ongoing [95,109,110,111,112,113,114,115,116,117,118,119,120,121,122,123,124]. The luminescence mechanism is explained by considering the quantum energy levels (bands) of materials, determined (i) by the structure of the crystal lattice and (ii) by the presence of appropriate impurities from other elements, called *activators* (Figure 4). E.g., thallium (Tl) in NaI (Tl), terbium (Tb), in Gd_2_O_2_S (Tb) etc. The basic crystal lattice, without an activator, is called a *matrix* or *host*. The presence of the activator ions causes distortion in the lattice at some points, called *luminescent centers*.

The energy levels involved in the process are divided into two distinct energy bands (Figure 4), i.e., the valence band and the conduction band, separated by a forbidden energy gap between. Bound electrons lay in the lower energy valence band. The absorption of ionizing radiation (X, γ radiation, etc.) results in the mobilization of a fast electron (e.g., photoelectron, Compton electron, e--e+ pair), which, moving through the material, transfers energy to electrons of the lattice. These electrons jump in the conduction band, leaving positive holes in the valence band. Each fast electron creates a large number of electron-hole pairs. Electrons arriving at a luminescent center may occupy its upper energy state and then deexcite to recombine with a positive hole, resulting in the emission of light photons. Often, however, a significant part of the energy of the fast electron is channeled into the development of phonons (vibrations propagating through the lattice, called phonons). For the creation of an electron-hole pair, an energy significantly higher than the energy gap (*E_g_*) is required. It has been estimated that the average W energy required to create a pair is (Equation (22)):

(22) $$W=\beta E_{g}$$

where *β* is a parameter with various values depending on the type of material. E.g., for NaI and CsI, it is *β* = 3, for CaWO_4_, used in conventional X-ray cassettes, it is *β* = 7, for rare earth materials such as La_2_O_2_S, it is *β* = 4, etc. The number *N_φ_* of optical photons produced within the mass of the scintillator-phosphor is given by (Equation (23)):

(23) $$N_{\varphi }=\frac{E}{\beta E_{g}}Sq$$

where *E* is the absorbed radiation photon energy, *S* is the transfer efficiency of an electron-hole pair to the luminescent center, and *q* is the quantum efficiency of the center. Most imaging systems using ionizing radiation are based on fluorescent materials, often referred to as *phosphors* or *scintillators*, used in various forms:

1. In the form of *screens*, i.e., thin coatings of a relatively large area (approximately 150–300 μm thick), used in intensifying screens integrated into radiographic and fluoroscopic detectors (radiographic cassettes, flat panel arrays of X-ray detectors, as well as portal imaging detectors). Screens are divided into two basic categories: *granular screens* and *structured screens*. Granular screens contain the fluorescent material (e.g., Gd_2_O_2_S) in granular form (grains of material dispersed in some binder). Due to the binder, the density of the active material (i.e., the *packing density*) is significantly reduced to about 50% of the nominal density of the material. These structures are often referred to as *phosphors*, although very often this holds for structured layers as well. In granular screens, optical scattering in the grains of the fluorescent material contributes to the attenuation (due to multiple scattering) of the fraction of laterally directed light. Multiple scattering lengthens the total path of the “sideways” photons, and this increases attenuation probability due to the exponential law of light attenuation. In this way, the light spreading, and the sharpness degradation of the light spatial distribution are avoided. Furthermore, the size (5–10 μm) and the shape of the grains affect both the screen sensitivity (QDE and light emission efficiency), which increases with grain size, and the imaging performance (MTF-spatial resolution), which degrades with size. An additional influencing factor is the thickness of the phosphor layer. Thick screens show higher QDE, while thin screens improve MTF. In fibrous (structured) needle-like crystals, the light is trapped through multiple reflections inside the fibers (cracks), and thus its spreading inside the material is avoided. These crystals improve significantly MTF-spatial resolution since light photons are trapped within needles and do not spread in the material’s mass. Thus, thick screens with increased radiation absorption can be prepared from such materials without spatial resolution degradation. The packing density of corresponding screens is of the order of 70–80%. Very popular materials of this form are CsI:Tl and CsI:Na, which have been largely employed in radiography, fluoroscopy, and portal imaging. In various screens, the number of light photons created per absorbed X-ray ranges from several hundreds to a few thousands (e.g., 800–4000), depending on the X-ray energy, QDE, effective atomic number, light attenuation, etc.

2. In the form of *single-crystals*, often referred to as *scintillators*, used: (a) as flat plates of large area and relatively small thickness; and (b) as numerous small rectangular area-thick crystals (such as rods). Large surface-area single crystals are used in nuclear medicine imaging systems such as planar γ-camera and SPECT or positron cameras (see Section 5.3). Small entrance area multiple single crystals, 10 to 30 mm thick, are used in ring-shaped nuclear medicine tomographic systems (mostly in PET, see Section 5.4). The number of crystals may be of the order of tens to hundreds of thousands (e.g., in total body PET scanners), with a cross section of 2 × 2 mm^2^ to 4 × 4 mm^2^ coupled to PSPMT, APDs, or SiPM optical sensors (see next paragraph). Single crystals are often used in CT detectors, arranged in an 2D array in arc shaped configuration, the thickness ranges from 0.3 to 3 mm and the cross section is of the order of few mm (see Section 5.2). A key characteristic of single crystals is their high optical transparency.

3. In the form of *transparent ceramics*, which are often used in CT detectors. These materials are in polycrystalline form, produced by various techniques (e.g., sintering of grains of nano-sized powders, generally followed by hot pressure). Ceramic materials may show higher emission efficiency than corresponding crystals and are of lower cost [99,118].

4. In the form of *storage phosphors* (based on the effect of *photostimulated luminescence*-PSL), which are used in X-ray computed radiography systems (see Section 5.1.3). After X-ray irradiating these materials, many electron/hole pairs are trapped in metastable states, i.e., they do not transfer their energy to a luminescent center (after recombination). Only if red or near-infrared photons interact with these states do the electrons absorb sufficient energy and escape to recombine with trapped holes and generate luminescence light. The photo stimulated luminescence is an anti-Stokes phenomenon since the emitted light photons have higher energy than the stimulating red photons. The energy required to create a trapped electron (and hole) is of the order of 60–100 eV, resulting in an amount of some hundreds (200–800) of trapped electrons per X-ray photon, depending on the application. In commercial CR systems, these phosphors are used in the form of large area screens (plates), consisting of phosphor grains, such as those in ordinary granular screens of conventional and digital radiography. Commercially used materials are BaFBr:Eu^2+^, CsBr:Eu^2+^, RbBr:Tl^+^ [31,115].

**Figure 5:**
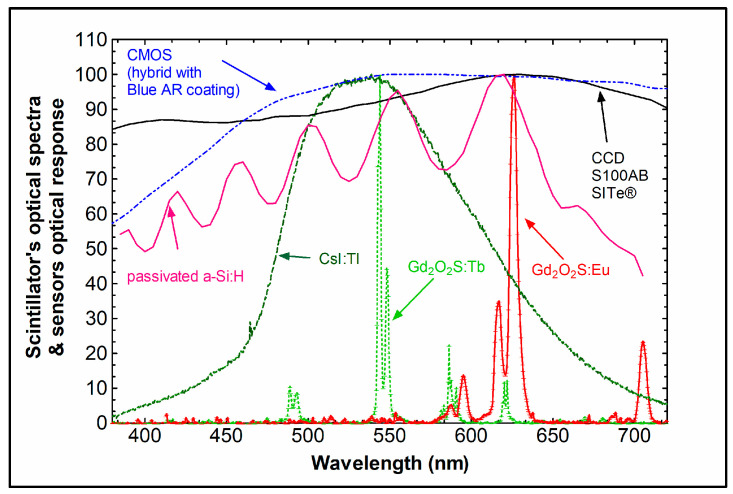
Light emission spectra and spectral sensitivity curves of various luminescent materials and optical sensors.

**Figure 6:**
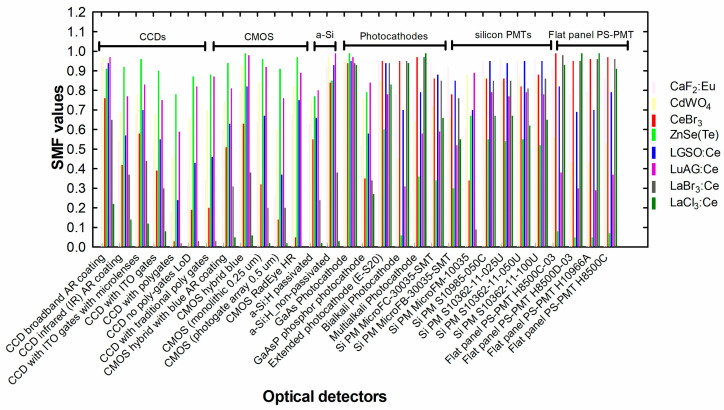
Spectral matching factors of various combinations of scintillators/optical sensors.

In addition to the aforementioned (see Section 3) radiation detection and conversion properties, the suitability of phosphor-scintillators for medical imaging can be assessed by the following additional properties [14,16,31,115]:

(i) The *light transmission efficiency* (LTE) and the corresponding spatial distribution of the light (PSF) at the emitting side of the scintillator. LTE corresponds to: (i) a quantum gain stage (g_3_), in the LCSA theory, equal to the fraction of generated light arriving at the scintillator’s emitting side; and (i) a blurring stage describing the spatial distribution of this light (stage T4 of an integrated system, see Section 2 and Section 5 and Figure 2), and depends on the transparency, index of refraction, the light scattering properties of the material, and the thickness of the particular scintillator sample. These properties have been analyzed in the context of Boltzmann’s diffusion differential equation as well as with Monte Carlo methods. It must be noticed that LTE has a blurring effect on both signal and noise. In transparent single crystals, mostly used in nuclear medicine and in CT, light created by a thin γ-ray beam is spread out in the whole scintillator mass. However, due to photon counting measurement techniques and, in some cases, to crystal pixilation, this may not be a problem. On the contrary, in these techniques, high transparency is required since it improves light collection by the optical sensors. In granular materials, light scattering reduces the extent of light spreading (PSF) by extinction of the laterally directed photons (due to elongated trajectories). This improves spatial resolution in X-ray radiography and fluoroscopy. In these methods, scintillator thickness may decrease transparency, light transmission, and emission, affecting similarly image quality [32,42,61,62,92]. In the spatial frequency domain, LTE is expressed by a corresponding MTF (see Section 2).
(ii) The spectrum of the emitted light (determined by the energy levels of the activator) and its compatibility with the quantum spectral sensitivity distribution of the optical sensor. This can be estimated by the *spectral matching factor*, *a*_s_, as follows (Equation (24)):

(24) $$a_{s}=\int_{\Delta \lambda }\phi_{p}\left(\lambda \right)S_{d}\left(\lambda \right)d\lambda /\int \phi_{p}\left(\lambda \right)d\lambda$$

where $\;\phi_{p}\left(\lambda \right)$ is the spectrum of the scintillator’s light and $S_{d}\left(\lambda \right)$ is the spectral distribution of the quantum efficiency of the corresponding optical sensor, Δλ is the light spectral range [32]. Figure 5 and Figure 6 show emission spectra of some scintillators with spectral distribution curves of various optical sensors. Values well above 50% are considered very appropriate.

(iii) *Decay time* τ (the time required for the light intensity to decrease to 1/e of the peak value), related to the probability of electric dipole radiative transitions between quantum states. It holds *τ ~λ*^2^ (λ: wavelength of light) [98]. According to this, scintillators with blue or ultraviolet emission should be faster. In X-ray fluoroscopy, in CT, and in nuclear medicine, short or very short (e.g., in PET) decay times are required. Data are shown in Table 2. In the case of more than one decay times, it holds: *n* = *ξ*_1_*exp*[−*t*/*τ*_1_] + *ξ*_2_[*t/τ*_2_] + … where *n* is the number of photons per unit of time,*ξ_i_* are parameters depending on the physical properties of the material and, in some cases, on the energy of the incident radiation, *τ_i_* are decay constants.
(iv) *Afterglow* i.e., emission with relatively longer decay, separated from the main emission. This can be from a few *ms* to some hours and may be a significant drawback for detecting materials,
(v) Natural radioactivity (from radioactive isotopes in the chemical composition, e.g., intrinsic radioactivity of LSO, LYSO, where Lu contains 2.6% of ^176^Lu).
(vi) Hygroscopicity (e.g., in NaI, CsI, etc.).
(vii) Fragility.

QDE, EAE, ICE, and LTE are principal factors related to the *luminescence efficiency* (LE) (=*Ψ_Λ_/Ψ_Χ_*, emitted light energy flux over incident X-ray energy flux:), which affects the brightness and the quality of images as well as the patient dose burden under clinical conditions. LE has been shown to be very high in Tb-activated phosphors. Regarding the decay time, Ce-activated crystals or powders, emitting blue light, exhibit a faster response. The Ce^3+^ ion emission originates from 5d level transitions to the 4f ground state (^2^F_7/2_, ^2^F_5/2_ levels) [98]. 5f → 4f transitions are allowed, and they produce emissions in broad spectral bands. Electron-hole pairs are rapidly transferred to the luminescent center, and hence very short decays are observed [125]. For Tb^3+^, the ^5^D_J_ → ^7^F_J’_ (e.g., ^5^D_4_ → ^7^F_5_) transitions have been observed [126]. Table 2 shows the properties of some popular phosphors.

### 3.2. Photoconductors

By definition, photoconductivity is the increase in electrical conductivity of a material that takes place when it is irradiated by photons. Photoconductors are used in direct conversion detectors [127,128,129,130,131,132,133,134,135,136,137,138,139,140,141]. Table 3 shows the properties of some commonly used photoconductor materials (together with semiconductors; see next paragraph). Advantages of using photoconductors are (i) fewer stages of signal conversion since conversion of radiation into light is not included in the signal transmission process and (ii) significantly lower signal spreading due to the applied electric field (see below). Disadvantages are the small variety of available materials and their forms (e.g., in comparison with scintillators), the relatively low radiation absorption index of the dominant material (see next), noise, and image lag. An index often used for photoconductors is the attenuation depth (δ), i.e., the depth of 63% attenuation of radiation. The material mostly used in these systems is amorphous selenium, with Z = 34, which is employed in the form of flat coatings of 130–500 µm (or more) thickness, placed in an electric field (e.g., for a field of 10 V/μm inside a 500 μm-thick layer of a-Se, a bias of 5000 V is required). The electric field is directed perpendicular to the plane of the coating. After the absorption of an ionizing photon, usually by photoelectric effect, an electron is ejected from the K-shell, which while penetrating the material’s mass creates electron-hole pairs and phonons. Phonons represent energy losses. This process is expressed by the following ratio giving the produced charge Q (Equation (25)):

(25) $$Q=\frac{eE}{\beta E_{g}}$$

where *E* is the absorbed photon energy and *β* is a parameter, which should be as low as possible; however, it has values higher than 2. The denominator of the above relation is often referred to as the energy *W (=βE_g_)* required to create an electron—hole pair, which is also expressed as (Equation (26)):

(26) $$W=W_{0}+BE^{-1}$$

where *W*_0_ is the intrinsic electron-hole creation energy (considered at infinite field) and E is the applied electric field. *B* is a parameter weakly depending on incident radiation energy. For a-Se, in the mammography energy range (20–40 keV), B is approximately 4.4 × 10^2^ eV.V μm^−1^. W_0_ is approximately 6 eV [134].

The electric charges then move along the field lines of the electric field and are driven to the surfaces of the planar coating. There they are collected by suitable electrodes. These are the same electrodes that create the electric field. One electrode is in the form of a thin flat plate and is placed on the upper face of the a-Se coating. The other electrode consists of a large number of individual discrete contacts. Through these electrodes, image formation is achieved. As mentioned above, the electric field is of the order of 10 V/μm but can reach up to 30 V/μm or even 80 V/μm. Increasing the intensity of the electric field causes a decrease in the transit time of the charges through the mass of a-Se. This improves the directivity of the collected electrons to the electrodes. It limits their spatial spread and thus causes an increase in the signal. The increased electric field can cause a reverse flow of charges that have opposite signs. That is, charge flow from and through the electrodes to the selenium coating. For this purpose, suitable blocking contacts are used (blocking contacts that prevent reverse flow).

Photoconductive materials are characterized by two physical properties: (i) they have a slightly increased forbidden energy gap, i.e., slightly higher than in semiconductors (of the order of 2.2 eV for a-Se). This limits the presence of free charges within the material before irradiation, and (ii) the lifetime of the electric charge carriers is sufficient to penetrate the mass of the material and reach its surface, where they are collected. That is, charges can travel long distances without getting trapped in the material’s lattice. The lifetime *τ* determines, together with the intensity of the electric field E, the average distance *S* that the charge carriers can travel (Equation (27)):

(27) S=μτE

*μ* is the *drift mobility*. The distance *S* (called the *schubweg*) must be sufficiently longer than the thickness of the a-Se coating (*S >> L*, *L* thickness of the photoconductive layer). The product *μτ* is the range of the charge carriers (schubweg per unit of electric field) [1,134]. Electrons and holes have ranges within the useful limits, within a-Se, and hence they can be easily collected. The values of *S* differ between electrons and holes. In a-Se for electrons it is 0.3–3 μm while for holes it is 6–65 μm (at an electric field of 10 V/μm). A key problem can be transverse conductivity. The term refers to the movement of charge carriers onto the surface of the a-Se coating and lateral charge propagation. It is clear that this phenomenon has a negative effect on the quality of the formed image. The consequences of this process are limited by additions of impurities that create charge traps near the a-Se surface. Amorphous selenium is characterized by a number of advantages and disadvantages. It has great uniformity with a corresponding impact on image quality. The manufacturing process (often physical vapor deposition—PVD) is low-cost, does not require high temperatures, and can be prepared in large dimensions. However, the atomic number (34) is relatively low, thus requiring thick layers for X-ray absorption. The properties of a-Se also show aging effects, i.e., some properties change with time, showing an exponential variation towards equilibrium values. Deterioration of some properties also occurs after irradiation over time, e.g., buildup of trapped charges may occur, causing recombination with new carriers. Other materials used as photoconductors are: PbI_2_, PbO, TlBr, and potentially: CdZnTe, CdTe, CdSe, and HgI_2_. Among them are some polycrystalline (granular) materials.

Photoconductors suffer from *dark current* (output electronic signal without radiation incidence), which should be the lowest possible (not exceeding 10 pA mm^−2^). Dark current is thermally generated and increases when the bandgap energy E_g_ decreases (which also increases photoconductor efficiency). Thus, a compromise between dark current (noise) and efficiency (or sensitivity) has to be made. Within this bandgap, various traps may be found that can trap and then de-trap charges, causing image lag and ghosting [134,135]. Resulting in carryover of image charge generated by previous X-ray exposures into subsequent image frames and changing of X-ray sensitivity, i.e., long-term image persistence. Recombination of newly generated charges with previously trapped ones may occur. This reduces the effective lifetime of mobile charge carriers and correspondingly affects X-ray sensitivity (ghosting). The mean drift length of the generated charges should be larger than the photoconductor layer thickness in order to avoid (or reduce) this image lag and ghosting.

Photoconductors are often evaluated by their X-ray sensitivity, which has been defined as follows [134] (Equation (28)):

(28) $$S_{x}=(\frac{5.45\times 10^{13}e}{{{(\mu }_{en}/\rho )}_{air}})\eta_{Q}(\frac{{{(\mu }_{en}/\mu )Ε}_{\lambda }}{W_{Ζ}})\eta_{cc}$$

An additional property of a-Se is the so-called *avalanche effect*, i.e., production of intense ionization effects under the influence of high electric fields (80 V/μm). The charge carriers obtain high kinetic energy and create new charges many times (by impact ionization) within the photoconductor mass. The efficiency to create charges increases by some orders of magnitude, and the charge collection efficiency is improved. The avalanche effect results when the holes in the a-Se mass gain energy faster than losing it to phonons (electrons are slow carriers and do not participate in the process) [132]. This effect has been applied in high-gain avalanche rushing photoconductor detectors (HARP).

### 3.3. Semiconductors

In semiconductors, ionizing radiation creates free electrons and holes in numbers proportional to the energy of the radiation. Semiconductors are used in a large variety of applications, from γ-ray spectroscopy to medical imaging [142,143,144,145,146,147,148,149,150,151,152,153,154,155]. The most common semiconductors used in radiation detectors are shown in Table 3. As it can be observed from data on the forbidden energy gap, the energy required to create charge carriers in the semiconductors is significantly lower than in scintillators (approximately 10 times). A larger number of charge carriers are created, and thus the statistical noise in energy determination is significantly lower, resulting in better energy resolution. In most cases, semiconductors are cooled to liquid nitrogen temperature (−195.8 °C) to avoid the contribution of thermally excited electrons to measurements. The density of free carriers (electrons-holes) created in a semiconductor is *n = Nexp*(−*E_g_/kT*)*,* where *N* depends on the density of energy states in the valence and conduction bands and *k* is the Boltzmann constant [142]. Semiconductors have generally faster response times than many scintillators. A good detector material must have high *resistivity* (>10^9^ Ωcm) to reduce dark current and other noise and a high, *mobility-lifetime product* (*μτ*). Under this condition, radiation-generated carriers can penetrate thick detector material to be collected by electrodes. Radiation detectors based on CdZnTe (cadmium-zinc-telluride-CZT) or on CdTe as well as on silicon (Si) are most often employed in medical imaging.

The nomenclature CZT refers to and denotes materials of the more general form Cd_1−x_Zn_x_Te. CZT can be considered as a solution consisting of CdTe and ZnTe. Variations in the value of x lead to physical property modification [148]. A key advantage of CZT and CdTe materials, in relation to germanium semiconductors (Ge:Li, HPGe), is that they are efficient radiation detectors at ambient temperatures. The term room temperature semiconductor detectors (RTSD) is often used to characterize these materials. They do not require cooling to liquid nitrogen temperatures. CZT and CdTe also have satisfactory radiation absorption coefficients with values very close to the corresponding values of the NaI scintillator crystal (due to the relatively high atomic numbers of Cd (Z = 48) and Te (Z = 52)). In addition, CZT and CdTe have a high density (5.78 g/cm^3^, 6.02 g/cm^3^, 6.20 g/cm^3^, for Zn_0.9_Zn_0.1_Te, Zn_0.8_Zn_0.2_Te, and CdTe, respectively), which is significantly higher than that of NaI (3.67 g/cm^3^) but also that of Ge (5.33 g/cm^3^) and Si (2.33 g/cm^3^). This contributes to increasing the radiation absorption and, therefore, the overall measurement performance of CZT and CdTe. The addition and adjustment of Zn result in strong covalent bonds between atoms and modify the energy bandgap, respectively, i.e., a larger bandgap allows for higher resistivity and lower leakage current.

The high resistivity is a significant advantage of CZT and CdTe (5 × 10^10^–10^11^ Ω.cm for both CZT and 10^9^–3 × 10^9^ Ω.cm for CdTe). The high resistivity is necessary because it limits the leakage current or dark current in the semiconductors. Leakage current is current that occurs without radiation incident on the material. This current is an important source of noise since the useful signal current due to the effect of radiation is weak (10^−6^ A).

Limitation of leakage currents has positive effects on energy resolution. The higher resistivity of CZT is due to the used crystal growth method (HPB-high pressure Bridgman technique) [152,153]. The addition of Zn also contributes to the increased value of the resistance, which increases the energy gap. CZT has a slightly larger energy gap than CdTe: it is Eg = 1.57 eV for Cd_0.9_Zn_0.1_Te, Eg = 1.5–2.2 eV for Cd_0.9_Zn_0.1_Te, and Eg = 1.47 eV for CdTe. However, the HPB development technique and the presence of Zn cause some problems related to the drift mobility of the holes. That is, additional traps and crystal defects are created that reduce the mobility of positive charge carriers (holes). The mobility (μ) of the electric charge carriers determines the speed (υ) at which the electric charge is collected from the electrodes (υ = μE, E: the applied electric field). Large mobility values reduce the charge collection time and increase the usable signal. The hole mobility within the crystal lattice of CdTe: Cl is higher than that of CZT (80 cm^2^/V.s vs. 50 cm^2^/V.s). On the contrary, the mobilities of the electrons are approximately the same in both materials (1000–1100 cm^2^/V.s). The absorption of X-ray or γ-ray photons in CZT, CdTe, creates electron-hole pairs (charge carriers). The energy required to create an electron-hole pair is well above the band gap energy. This is because an amount of energy is lost in phonons and heat. The number of charges created inside the material is proportional to the energy of the absorbed photon. The charge is collected using electrodes suitably designed in order to determine the coordinates of the x or γ ray absorption point. One useful parameter used to evaluate detectors is the *charge collection efficiency* expressed through the so-called Ramo theorem and the Hecht equation (Equation (29)):

(29) $$\eta \left(x\right)=\frac{\lambda_{e}}{L(1-\exp\left(-\frac{L-x}{\lambda_{e}}\right))}+\frac{\lambda_{h}}{L(1-\exp\left(-\frac{-x}{\lambda_{h}}\right))}$$

where $\lambda_{e}$ and $\lambda_{h}$ are the mean free paths of electrons and holes. *L* is the detector thickness. *x* is the depth within the detector at which the photon has interacted [153]. The mean free path is the average length of the path that a charge travels until it becomes trapped and is proportional to the velocity. The charge collection efficiency together with the absorption quantum efficiency and the geometric efficiency (1/d^2^) determine the overall efficiency of the detector array. Since the mobility of electrons is significantly higher than that of holes, the anode electrodes are of higher importance than the cathode ones in a charge collection device. In general, RTSDs show very good statistics of charge carriers, and this results in lower uncertainty in the measurements. Accordingly, this affects the energy resolution (e.g., <4.5%) in nuclear medicine detectors, which is better than the energy resolution obtained by scintillator detectors (>9.5%) [150].

A drawback of CZT is the so-called polarization effect, i.e., trapped charge accumulates and causes a build-up effect, creating an intrinsic electric field opposing the external applied field [156,157].

**Si detectors.** Si is used in the so-called *silicon drift* detectors, working as photon counting devices in digital mammography and in computed tomography [158,159]. Si converters have a low atomic number; however, they exhibit very good energy resolution; their K-fluorescence emission is at low energy, and this may be an advantage for some applications; they are of high purity with good collection efficiency [33]. 

Summarizing the properties of radiation-converting materials, we conclude that:

Phosphors and scintillators remain the most widely used radiation converters. Advantages of these materials are the huge variety of chemical compositions and forms, with various density and effective atomic number values (in some cases significantly higher than other materials), various intrinsic characteristics (energy gap and radiation conversion efficiency, optical attenuation coefficients, etc.), activated with various activators contributing significantly to the overall emission efficiency and decay times varying across a wide range of values, covering the needs of many applications. There is also a large variety of forms, e.g., granular materials, structures of needle-like crystals, single crystals, ceramic materials, organic materials, photostimulable crystals, and even quantum dots. Disadvantages of scintillators are the non-direct conversion of radiation into electric charge as well as the light spread that degrades spatial resolution. Research for new scintillators is ongoing and expanding, with new forms of materials (nanophosphors, scintillating fibers, quantum dots, micrometer-thick films, liquid-phase perovskites, etc.) being targeted. New materials such as the LFS-3 with excellent timing performance CRT are investigated. Based on these data, we conclude that scintillators can cover a wide range of applications [94,125].

Semiconductors have the advantages of creating a large number of charge carriers, resulting in high sensitivity (electrons per radiation photon) and very good energy resolution, and of directly converting radiation into electrical charge. Among them, CZT and CdTe are particularly promising materials. However, although heavy materials have been investigated, common commercially used semiconductors have, on average, a lower atomic number than scintillators.

**Table 3:** Materials for direct conversion detectors (photoconductors, semiconductors) [148]. Z: atomic number, ρ: density, E_g_: forbidden energy band gap, R: resistivity.

Material	Ζ	K-Edge (keV)	ρ (g/cm^3^)	E_g_ (eV)	I (eV/Ionization)	R (Ω)	μ_e_τ_ε_ (cm^2^/V)	μ_h_τ_h_ (cm^2^/V)
a-Se [134]	34	16.53		2.2			3 × 10^−7^–10^−5^	10^−6^–6 × 10^−3^
HgI_2_	80/53	148.39/51.88	6.4	2.13	4.2	10^13^	10^−4^	10^−5^
PbI_2_	82/53	158.6/51.88	6.2	2.3–2.6	4.9	10^12^	10^−6^	10^−7^
TlBr	81/35	4.96/13.47	7.56	2.68	6.5	10^12^	10^−5^	10^−6^
Si	14	1.83	2.33	1.12	3.62	10^4^	>1	1
Ge	32	11.1	5.33	0.67	2.96	50	>1	>1
CdTe	48/52	26.7/31.81	6.20	1.44	4.43	10^9^	10^−3^	10^−4^
Cd_0.9_Zn_0.1_Te	48/30/52	26.7/9.65/31.81	5.78	1.57	4.64	10^10^–10^11^	10^−3^–10^−2^	10^−5^
Cd_0.8_Zn_0.2_Te	48/30/52	26.7/9.65/31.81	6.02	1.5–2.2	5.0	10^10^–10^11^	10^−3^	10^−6^–10^−5^

## 4. Optical Sensors

Optical sensors (or photodetectors) are employed in indirect conversion medical imaging systems to convert light into an electronic signal [160,161,162,163,164,165,166,167,168,169]. Most of them are based either on photodiode (semiconductor p-n junction) technology or on photocathodes. Optical sensors also include photosensitive silver bromide emulsions (film), phototransistors, photoresistors, etc. In most cases, optical sensors consist of an array of sensor elements of small dimensions, often referred to as “aperture”.

Light absorption is due to quantum mechanical transitions of electrons from lower energy states to higher ones. This transition may be from the valence to the conduction energy bands of a material or from the valence band to the continuum (i.e., emission of a photoelectron according to Einstein’s photoelectric effect). In the first case, charge carriers are created, which either decrease the resistance (*photoconductive mode*) or a voltage across the depletion region is generated (*photovoltaic mode*). In the second case electrons are liberated (*photoelectric mode*) and can be accelerated and focused by electric fields [160].

Some basic characteristics of optical sensors are: (a) the *quantum efficiency* (QE) or; *(b)* which is generally defined as the number of photoelectrons released per incident optical photon. QE is also called *photoefficiency*. Another relative quantity used is the *photosensitivity* expressed in *μA/lm* (microamperes/lumen). That is, the delivered electron current in *μA* to the incident luminous flux in lumens. Similarly, the *responsivity* (R) is defined as the output current over the incident light power. Responsivity is related to quantum efficiency by the relation *QE = R*(*hν/e*). *Gain* is a similar metric expressing the output current over the initial photocurrent generated by light photons. *Photon detection efficiency* is also defined in particular cases (see SiPMs), relating quantum efficiency, spectral response, geometric factors (i.e., fill factor), etc.; (b) *Noise equivalent power* (NEP) is the amount of light required to obtain SNR = 1 (signal power over noise power) [160]. NEP is inversely proportional to *detectivity,* which is defined as the square root of the detector’s area over NEP and corrects for the dependency of NEP on detector area; (c) the spectral response or spectral sensitivity, which expresses the change of the quantum efficiency in relation to the spectrum of the incident light, as well as the spectral compatibility, which expresses the coincidence of the spectral response of the photodetector and the spectrum of the light emitted by the scintillator; (d) the *response time* or *rise time,* which is the time required to attain 90% of the response, measured from 10%; (e) the index of refraction, which expresses the phenomena of optical refraction and optical reflection at the interface between the scintillator and the photodetector; (f) dark current or dark counts: it is an electrical signal at the output without any form of radiation falling on the photodetector [153,164]; (g) *Thermal noise,* which is due to thermal agitation of electrons in a resistance and is given as *S(f)* = 4*kT*Δ*fR*, where *S*(*f*) is the noise power spectrum, *T* is the temperature, *R* is the resistance, and Δf is the noise bandwidth. In many cases, the dimensions of the elements of optical sensor arrays (e.g., active matrix of photodiode arrays, CCDs, etc.; see Section 4.3 and Section 4.4 for instance) define the “aperture” that corresponds to a deterministic blurring stage, according to LCSA. This is expressed by an MTF, i.e., T_6_ in Figure 2.

Signals from analog output optical sensors are digitized by high-speed analog to digital converters (ADC) and then fed to read-out electronics. Application-specific integrated circuits (ASIC) that offer various functionalities are employed to read out the electronic optical sensor output. Field programmable gate arrays (FPGA) are also used in some systems. ASIC chips are based on metal oxide semiconductor (MOS) integrated circuit technology and are designed for a particular use. FPGAs are not application-specific since, due to programmable blocks, the same FPGA can be used for various applications.

### 4.1. Photocathodes

Photocathodes are thin layers prepared from materials exhibiting low ionization potential (binding energy B). They absorb light and emit photoelectrons according to the photoelectric effect (E_e_ = hν − Β). They are located at the input of photomultipliers or image intensifiers. Photocathodes may be made of a thin transparent layer of cesium—antimony (Cs-Sb) or cesium—scandium (Cs-Sc). A frequently used material is the dialkali SbK_2_Cs, whose spectral sensitivity is compatible with the wavelength of NaI (Tl) photons. There are also “trialkaline” type photocathodes, e.g., SbKNaCs, as well as Cs_3_Sb photocathodes. The last two are characterized by high sensitivity. When activated by cesium, materials of group V–III may exhibit negative electron affinity and thus negative binding energy [153]. When light (or any other form of radiation) hits the photocathode, electrons (photoelectrons) are emitted and may be then accelerated and focused to hit a target, i.e., the rear screen of an image intensifier or the dynodes of a photomultiplier. The quantum efficiency of the photon-to-electron “conversion” process is a function of the electron “extraction work”. Typically, 1 to 3 photoelectrons are emitted for 10 incident optical photons.

### 4.2. Photodiodes

**Figure 7:**
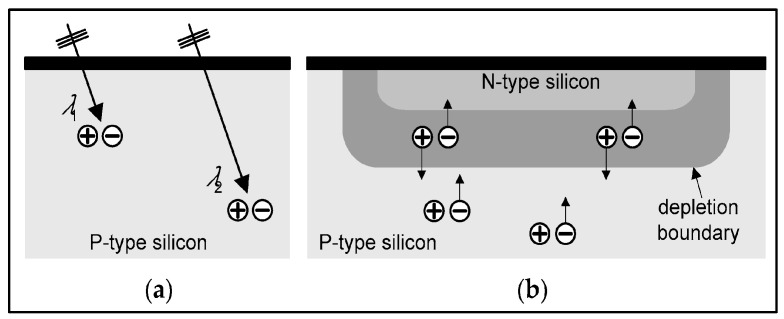
Photodiode: (**a**) Different wavelength photons create electron-hole pairs at different depths within the semiconductor material; (**b**) pn junction, n-type silicon, p-type silicon, depletion region, and electron-hole pairs [164].

Photodiodes (PD) are devices with a p-n or a p-i-n junction that convert optical photons into electrical current. The p-n junction is formed when p- and n-type semiconductors come into chemical contact, resulting in a potential difference between them (Figure 7). Electrons from the n region are directed towards the p region, where they recombine with the holes. In this way the so-called depletion region is formed, where charge carriers have been moved away by diffusion, forced by the internally created electric field. This region is also affected by the electrical voltage, which can be applied to the ends of the two semiconductors (bias). A common bias of the p-n contact is the so-called inverse bias, i.e., the n-type semiconductor is connected to the positive pole of a DC voltage source and the p-type semiconductor to the negative pole. This increases the potential difference across the pn junction. Also, the range of the depletion region increases. Inside this region, a strong electric field prevails because of the lack of carriers (i.e., electron-holes that have recombined). This area is the active or sensitive detecting area.

Photons interacting in the photodiode create charge carriers (electron-hole pairs). If this happens in the depletion region (or nearby), electrons and holes move towards the corresponding poles (anode-cathode), thus creating a photocurrent. Photodiodes can be prepared from a variety of materials, including silicon, germanium, and indium gallium arsenide. For silicon, which is used in most cases, the effective optical bandgap is 1.1 eV with a light absorption coefficient around 104 cm^−1^. This facilitates light absorption. In p-i-n junctions a highly doped intrinsic layer is inserted between the two layers (p and n). The intrinsic layer in p-i-n junctions, is highly resistive and increases the electric field strength. The depletion region in the photodiode is significantly increased, and the capacitance of the junction is decreased. This increases the speed of the photodiode. The increased layer also allows for a larger volume of photon to electron-hole conversion and higher quantum efficiency. The whole process is based on the so-called photoconductive mode of operation. The photocurrent developed in the photodiode is given as [164] (Equation (30)):

(30) $$i_{ph}=e\eta_{QE}P_{i}/hv$$

where η_QE_ is the quantum efficiency, P_i_ is the energy flux (power) of the incident light, h is Planck’s constant, ν the frequency of light photons, and e is the electron charge. The responsivity (Equation (31)):

(31) $$R=i_{ph}/P_{i}=e\eta_{QE}/hv$$

**Figure 8:**
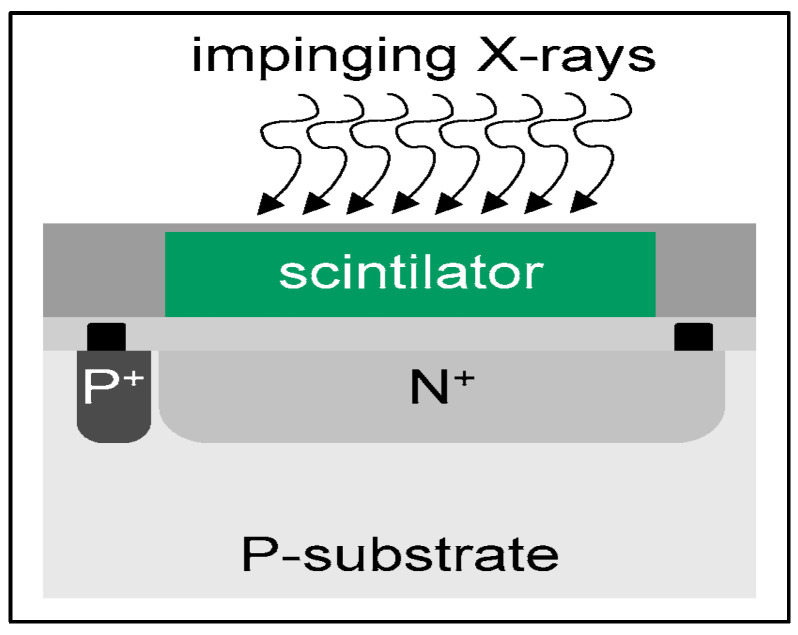
Detector element containing a scintillator on top of a N^+^ well—P-substrate [164].

Figure 8 shows an indirect conversion X-ray imaging detector element consisting of a scintillator layer on top of a N^+^ well-Psub photodiode (N^+^ denotes N-type semiconductor with high doping concentration; a n-well in a p-type doped semiconductor substrate; is a localized part of the substrate that has been doped with n-type atoms or molecules, making this part of the semiconductor n-type).

In the photovoltaic mode, no reverse bias is applied (unlike in the photoconductive mode, where the n-type semiconductor is driven positive with respect to the p-type semiconductor), and neither is normal bias. The collection of charges is due to the intrinsically developing potential difference between the two ends of the photodiode. If reverse bias is applied (photoconductive mode), some leakage current—dark current—would occur. This would cause the noise level to increase.

**Figure 9:**
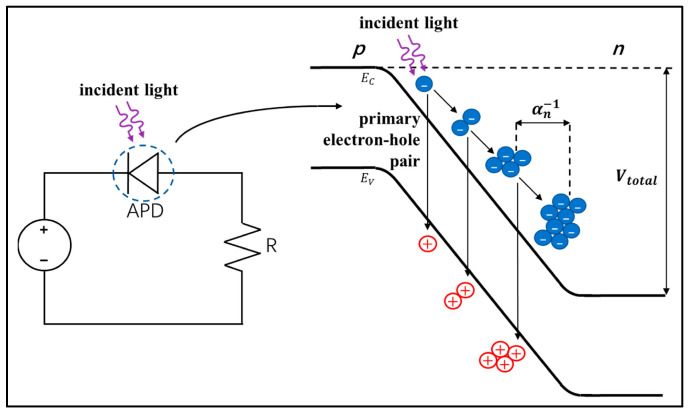
Potential across the pn junction EV valence band, EC conduction band. The impact ionization process. Here, $\alpha_{n}^{-1}$ is the average distance between each electron multiplication event, while *Vtotal* is the applied reverse bias plus the built-in potential [161].

*Avalanche photodiodes* (APD) are operated under conditions of near reverse bias breakdown voltage, creating an internal gain by avalanche effect (Figure 9). Carriers generated by photons produce more pairs, and so they are multiplied in the form of an avalanche breakdown (impact ionization). This creates an internal gain (usually of the order of 0.5–1.5 × 10^2^) within the photodiode, which in turn increases the current per photon. Gain (or multiplication factor) has been expressed as [160] (Equation (32)):

(32) $$G=\frac{\left[1-\frac{a_{p}}{a_{n}}]exp[a_{n}L(1-\frac{a_{p}}{a_{n}})\right]}{1-(\frac{a_{p}}{a_{n}})exp[a_{n}L(1-\frac{a_{p}}{a_{n}})]}$$

where *L* is the depletion region width, a_n_ and a_p_ are the impact ionization coefficients, defined as the reciprocal of the average distance traveled by a carrier before generating an additional electron-hole pair (for a given electric field). Gain can be as high as 10^3^; however, it is kept at a significantly lower level due to increasing noise. The latter is higher than that of simple photodiodes; however, this is compensated by the higher signal-to-noise ratio. APDs have been used in PET detectors, where their reverse voltage is appropriately adjusted to work under proportionality mode, i.e., the output signal being proportional to the total amount of incident light. Gain variation with voltage is linear and is affected by temperature changes. The maximum size of APDs is about 1 mm^2^ [170,171]. The spectral sensitivity ranges between 300 and 1100 nm.

### 4.3. Charged Coupled Devices-CCD

**Figure 10:**
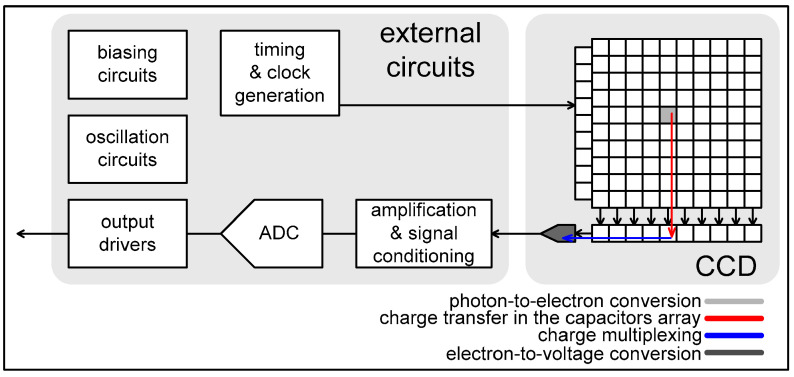
CCD array and read-out electronics [164].

Instead of simple photodiodes, arrays of charge-coupled devices (CCD) are often used in some DR and digital mammography detectors as well as in γ-camera units [6,12,19,164,172]. These arrays are integrated circuits of metal-oxide-semiconductors (MOS) and consist of 1. A series of metal electrodes called gates, 2. A layer of silicon oxide (SiO_2_) on which the metal electrodes are deposited, 3. A silicon semiconductor (e.g., p-Si) substrate that is under the SiO_2_ layer. MOS structures act as capacitors, which are charged up by the photogenerated charges. One armature of the capacitor is the respective gate electrode, and the other armature is the opposite surface of the silicon. Silicon oxide acts as an insulator-dielectric. After applying voltages in the gates, depletion regions are created in the silicon, and thus in each MOS, a potential well is developed, which acts as a charge trap. The depth of the well depends on the voltage applied to the corresponding electrode (gate). By suitably modifying the voltages, charges are transferred from one capacitor to the next in the array (from one well to the next well) (Figure 10).

MOS capacitors are also called picture elements (pixels) or detector elements (dels). In an CCD array, several rows of capacitors are placed next to each other. There are CCDs with 256 × 256 pixels up to 2048 × 2048 pixels. The dimensions of each pixel are of the order of 15 μm to 100 μm.

When photons from the scintillator hit one of the capacitors, electrical charges appear on the silicon due to the photoelectric effect. Electric charges are then transferred to the adjacent capacitor, where the potential is higher (“deeper well”). In this way, the charge is transferred from one capacitor to the next, i.e., to a deeper and deeper well. Finally, the charges are transferred at the output of the device. The transfer process is characterized by the so-called *charge transfer efficiency* (fraction of charge transferred), which is given as (Equation (33)):

(33) $$\eta_{TR}=\prod_{i}\eta_{i}$$

where *η_i_* is the fraction of transferred charge of pixel, which is lower than unity, i.e., increased MOS elements decrease the overall efficiency. However, CCDs retain high resolution with respect to other sensors. They have been used in digital mammography systems, where they are coupled to scintillators via fiber optic tapers, lenses, or, in some cases, by direct deposition of a scintillating screen on a CCD array. Optics (fibers, lenses) may affect overall system noise performance due to optical photon losses.

CCDs have a fill factor (active area over total area) of the order of 100%, resulting in high quantum efficiency and small pixel size (20–25 μm), which in turn ameliorate spatial resolution. They exhibit a linear response, wide dynamic range (65–70 dB), and rather low read noise (20 electrons), which however may be a problem in applications requiring high frame rates (due to charge transfer from pixel to pixel).

A drawback of CCD systems is related to read noise, i.e., low-level signals cannot be discriminated, since they may be of the same order as read noise. This can be a limiting factor for applications requiring high frame rates at low light fluence. To avoid this problem on chip, electron multiplication gain technology has been incorporated before final signal amplification (before read noise), thus creating the so-called electron multiplication CCDs (EMCCD) in an all-solid-state sensor. Another drawback of CCDs is that it is impossible to integrate the read-out electronics in one chip, as in CMOS sensors (see next paragraphs).

### 4.4. Active Matrix Array (AMA) Flat Panel

**Figure 11:**
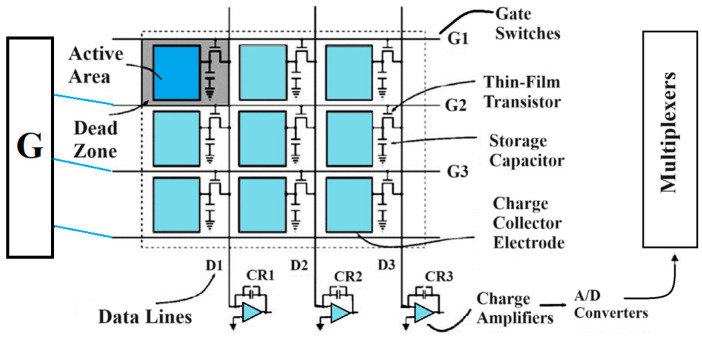
Active-matrix flat panel imager with amorphous silicon photodiodes and thin film transistors (TFT). G: gate drivers. Adapted from [174].

**Figure 12:**
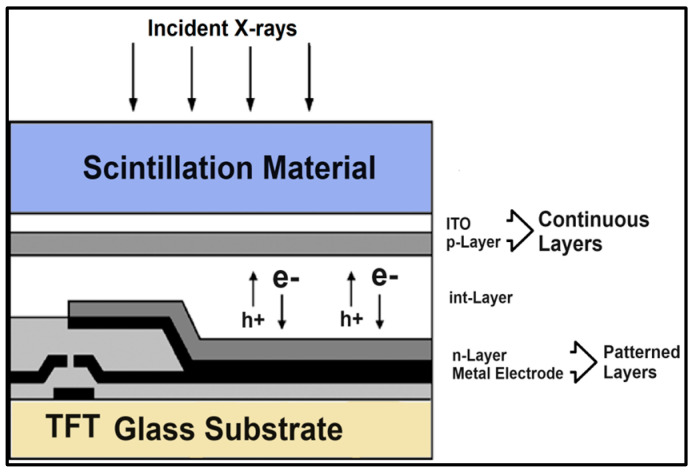
Digital radiography detector element. Adapted from [70].

This sensor is widely used in projection X-ray imaging (DR, DF, FFDM, portal imaging). It is also used in some CT systems [1,3,12,13,19,66,68,69,70,71,134,135,173]. An array of detector elements (dels or pixels) is placed on top of a glass base (Figure 11). Each element (Figure 12) consists of a reverse biased amorphous hydrogenated silicon (a-Si:H) photodiode (n-i-p/p layer, int layer, n-layer) connected to a thin film switch, i.e., a TFT (thin film transistor), FET (field effect transistor), or a diode or a pair of diodes. TFTs are also made from amorphous silicon (a-Si:H) or from polycrystalline cadmium selenide (CdSe) and, more rarely, from crystalline silicon. Layers of very large area, covering the requirements of X-ray projection imaging, can be prepared from a-Si, and this is a principal advantage of this material. Additional properties are the possibility of preparing thin films with low reverse leakage current. Hydrogen occupies many crystalline errors (dangling bonds) in the a-Si structure. In the case of indirect conversion detectors, the a-Si photodiodes absorb light from the phosphor-scintillator (with a light absorption coefficient of the order of 105 cm^−1^, for a-Si, which is slightly higher than that of c-Si), create electrons by the external photoelectric effect, and then act as charge capacitors. The flat photodiode array of the elements has large dimensions (e.g., 30 × 40 cm^2^ with 2904 × 3200 elements, 43 × 43 cm^2^ with 3000 × 3000 elements, 25 × 45 cm^2^, 45 × 45 cm^2^, etc.) and is called an *active matrix*. The elements measure 143 mm to less than 100 mm. The fill factor may be around 75%. On top of this array of photodiodes and TFTs, there is either a scintillating screen or a coating of photoconductive material, e.g., an amorphous selenium (a-Se) plate [134].

A transparent conductive indium-tin oxide (ITO) electrode is attached to the surface of the photodiode facing the scintillator. This electrode is biased at about 5 V. A relatively thick intrinsic (i) layer is sandwiched between the top p-doped and bottom n-doped layers of the photodiode. This layer interacts mainly with the optical photons of the scintillating screen. The n and p layers are very thin. Care must be taken to avoid loss of optical photons in these layers. On the other surface of the photodiode, there is another electrode connected to the drain of the TFT transistor. TFTs, acting as switches, are initially in a nonconductive state. During this state, the charge, i.e., the useful signal, accumulates in the photodiode. The charge causes a negative electrical voltage to develop across the ends of the TFT. This voltage should not exceed the magnitude of the voltage applied to the gate of the TFT to keep it in a non-conducting state. A positive gate voltage of +10 to +15 V is used to switch the device on and a negative voltage of −5 to −10 V to switch it off. This is achieved through appropriate gate driver circuitry. These circuits control the TFT gates via gate lines. Each horizontal row of TFTs corresponds to a gate line (Figure 11), which feeds all the TFTs in the row. Therefore, as many gate lines are available as there are rows of the active matrix. This phase of detector operation is called signal integration. The next phase is the signal readout phase, in which the gate driver circuits make the TFTs conductive. Each time a row of TFTs becomes conductive. Then the charges from the photodiodes are channeled, through the TFTs, to the so-called data lines, which transfer them to preamplifiers and from there to multiplexers. That is, the signals from the photodiodes located along a horizontal row end up in a parallel way at the inputs of the multiplexer. These signals then exit the multiplexer in sequence and are routed to analog-to-digital converters. The next step in the process is to repeat the described process on the elements of the next row.

The operation of direct detection systems is similar. In these systems, the photodiodes simply act as additional storage capacitors. This happens because amorphous selenium converts X-rays directly into electron-holes and, at the same time, has the role of a capacitor (see related Section 3.2 and Section 5.1.1).

AMFP sensors show low reverse leakage current and thin-film structure; they can be prepared in large areas for projection imaging and show good general performance; however, they have relatively low DQE at low exposure levels. Under particular circumstances (high frame rates), they show an excess of image lag, ghosting, and baseline drifts due to the amorphous structure of silicon (Si) or selenium (Se) [72,175].

### 4.5. Complementary Metal Oxide Semiconductor—Active Pixel Sensor (CMOS APS) Arrays

**Figure 13:**
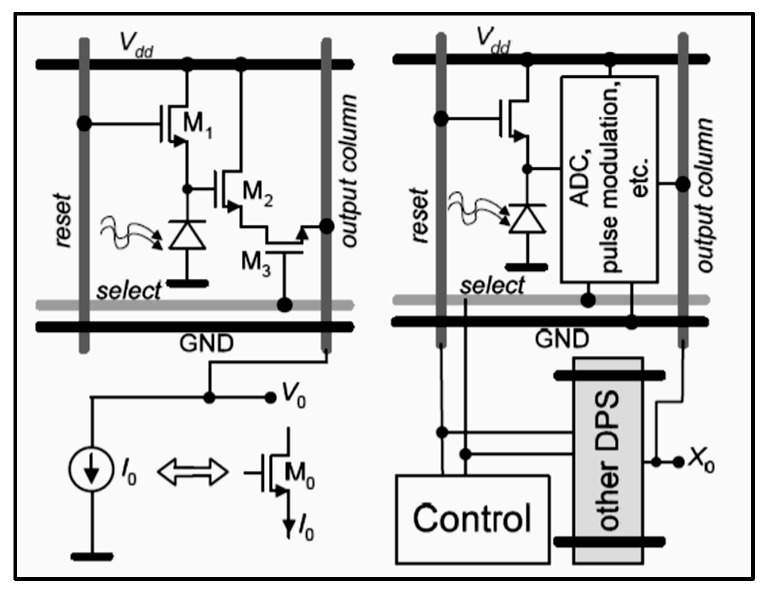
Active pixel sensor CMOS; shown are the photodiode and the three transistors [164].

**Figure 14:**
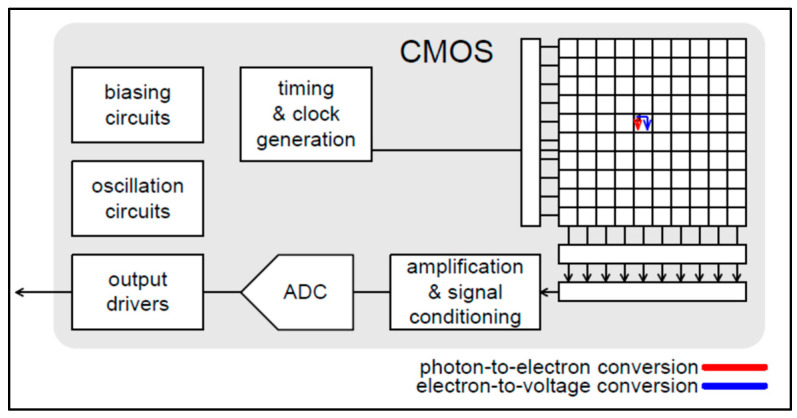
CMOS array and read-out electronics in one chip [164].

CMOS APS are systems on chip integrating sensors incorporating ADC (Figure 13 and Figure 14) [72,73,162,163,164,168,169,175]. CMOS array sensors are newly introduced in DR, DF, FFDM, PET, etc. They consist of a large array of sensor elements (or pixels) with 25–100 μm pitch. Elements contain a crystalline silicon (c-Si) photodiode and three MOSFET transistors in each element: a transistor that resets the photo-sensing element (reset transistor), a transistor to convert the accumulated charge (created by incident light) to voltage (source follower input transistor), and a transistor to select the row to be read (row select transistor) [176]. Regarding medical imaging, CMOS APS arrays of approximately 10 cm can be tiled together for larger detector areas (e.g., 20 × 30 cm^2^), e.g., for digital projection imaging. Pixel arrays larger than 2500 × 2700 have been achieved. CMOS APS are based on the determination of the charge accumulated on the photodiode (working as capacitance), i.e., they work as integrating sensors. This sensing element consists of an n-well to p-substrate photodiode. The operation of these circuits is to reset the photoelement, allowing charge to accumulate and then sensing the charge value. They are characterized as “active” in the sense that an active amplifier is included within each pixel (source follower input transistor). The readout is column-parallel with adjustable gain column amplifiers and a 10-bit single-slope ADC (integrated into the sensor). For the pixels in each row, simultaneous data conversion is achieved. For a given brightness, the slope of the ADC may be adjusted so that the best dynamic range is attained. All the signals for the control of the read-out hardware were created on-chip. All of the bias currents and voltages are generated on chip by a programmable DAC. Readout may be achieved by specifically designed field programmable gate array (FPGA’s) acquisition hardware.

Compared to CMOS-PPS, CMOS-APS have some improved properties: (i) the smaller pixel pitch (25–100 μm), (ii) the lower electronic noise (50–300 e^−^), (iii) fast frame rate [up to 30 frames per second (fps)], and (iv) full circuit integration [72,164,175]. General advantages of CMOS sensors are the well-established fabrication process, the low cost, the low power consumption, the system-on-chip capability, the small size, speed, etc. However, CMOSs exhibit relatively high dark current [164]. Active pixel sensors based on amorphous In–Ga–Zn–O (a-IGZO) TFT technology, which can offer high mobility (5–20 cm^2^ V^−1^ s^−1^) and low leakage current, have been proposed for use in digital tomosynthesis detectors using amorphous selenium as an X-ray converter [169].

### 4.6. Photomultiplier Arrays

**Figure 15:**
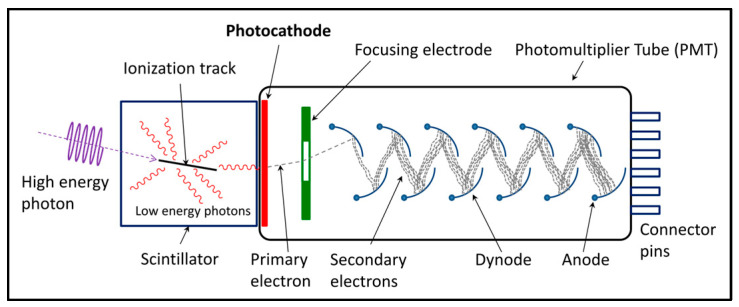
Photomultiplier tube [177].

These devices are principally used in nuclear medicine. A photomultiplier array consists of a number (e.g., 4–100) of photomultiplier tubes (PMTs), i.e., vacuum-tube based electronic devices that absorb light pulses and convert them (one by one) into electrical pulses (consisting of a large number of electrons) [2,4,9,17,34,160,161]. This is achieved: (a) with the photocathode, a coating with a low electron work function, located at the entrance of each photomultiplier, that absorbs light energy and emits electrons, with a quantum efficiency of 0.35; and (b) with dynodes, i.e., electrodes at a gradually increasing electrical potential, accelerating electrons (Figure 15). Dynodes, which are coated with a low-work function material, absorb the accelerated electron’s energy and re-emit a multiple number of low-energy electrons. That is, apart from the conversion of light into electrons, the photomultiplier greatly increases (multiplies) the number of electrons that make up a pulse. The sensing and accelerating structure of the PMT are enclosed in a vacuum tube. The number of electrons at the output is given as (Equation (34)):

(34) $$N_{T}=N_{\varphi }({Ε}_{\gamma })TG\eta_{ce}C_{S}A^{n}$$

where *N_φ_* is the number of incoming optical photons, *E_γ_* is the energy of the γ-ray photon, *T* is the transparency of the scintillator crystal, *G* is the geometric collection efficiency, i.e., optical photons arriving over those emitted by the scintillator, *η_ce_* is the quantum efficiency of the photocathode (electrons per photon), *C_s_* is the spectral compatibility (matching), factor and *A* is the multiplication factor or secondary emission factor. A (number of secondary electrons emitted by the dynode to the number of initial electrons incident on it), n is the number of dynodes. *A* depends on the potential difference U (100–200 V) between the dynodes (Equation (35)):

(35) $$A=\xi U^{a}$$

where *ξ* is a constant and α is a quantity that depends on the dynode shape and coating material. *A^n^* is of the order of 10^6^.

*Position-sensitive photomultipliers (PS/PMT).* PS/PMTs have the ability to determine the point of light incidence on the surface of the photocathode. Such photomultipliers are used in most PET systems as well as some newer SPECT systems. They differ in the structure and manufacturing characteristics of the anode and dynodes. There are two basic types of anodes: (a) cross-plate anodes or multiwire anodes, and (b) the multi-anode type. The first type of anode consists of many horizontal and vertical thin crossed metal strips (cross plate anodes). These strips correspond to the x and y directions and can be 4 × 4, 6 × 6, 18 × 18. The distance between the strips is of the order of very few millimeters. By recording the strip outputs, it can be determined which horizontal (x) and which vertical (y) wire provides the strongest electronic signal. The point of “intersection” of the strips corresponds to a small region in the photocathode receiving the photons. It should be noted that often the photocathode is square.

**Figure 16:**
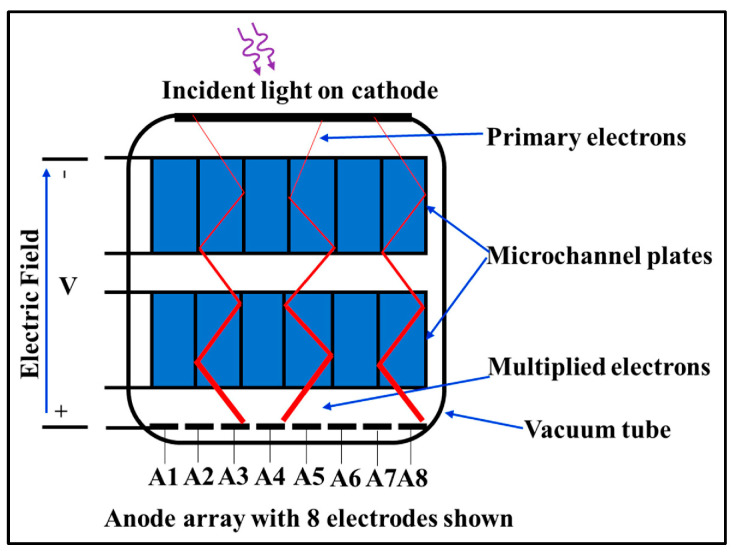
Position-sensitive photomultiplier tube with multichannel plates [161].

The second type of anode (multiple anode) consists of several discrete sections (plate electrodes). These sections make up a flat parallelogram or circular arrangement of elementary anodes (Figure 16).

The total number of individual anodes can be from 4 to 64 (8 × 8). A class of position-sensitive photomultipliers is the so-called *multi-channel plate* (MCP). This type consists of consecutive flat plates that have several hundreds of small channels (3–10 μm) inside them (Figure 16). Along the microchannels, which are coated by a photo-emissive material, electron beams are accelerated and multiplied. The intensities of these beams maintain the original photon intensity distribution incident on the plane of the photocathode. Compared to solid-state photodiode-based sensors, PMTs show lower dark current, principally due to their vacuum technology; they also show significantly higher gain and temporal resolution.

### 4.7. Geiger Mode APDs and Silicon Photomultipliers (SiPM)

**Figure 17:**
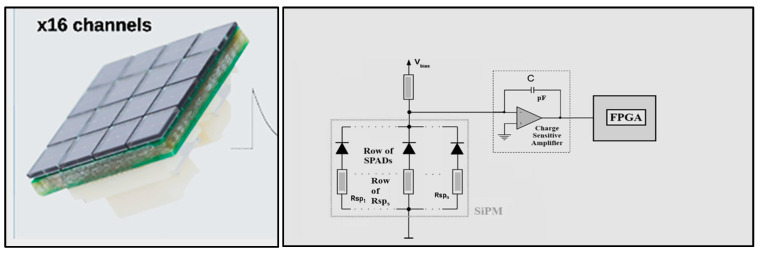
Array of a 4 × 4 SiPM and equivalent circuit of SiPM. A single pixel is a series combination of an avalanche photodiode and a quenching resistor. All of the pixels are connected in parallel [183].

Ordinary APDs may have limited gain and poor timing resolution (~2–3 ns) for some applications (e.g., TOF PET). Geiger-mode APDs (G-APD) or single-photon APDs (SPAD) are relatively free from such limitations. Geiger-mode avalanche photodiodes (G-APDs) consist of an array of microcells, each of which constitutes an avalanche photodiode-APD. These photodiodes are biased at a voltage higher than their breakdown voltage. This is the voltage above which general ionization occurs, with secondary, tertiary, etc. ionizations (self-sustaining impact ionization). That is, with the absorption of an optical photon, an avalanche discharge is caused, similar to that seen in Geiger-type detectors (i.e., gas ionization detectors). For this reason, this state is called Geiger state or Geiger mode. Often G-APDs are also called *silicon photomultipliers* (SiPM) due to their great ability to multiply electric charge carriers (electrons-holes), e.g., by 10^5^–10^7^ times with a bias of 50 V (which is significantly lower than that of PMTs) [161,164,165,166,167]. Even for a single incident light photon, they can generate a large output signal. SiPMs are not susceptible to magnetic fields (better than APDs), and in general, they combine the properties of both APDs and PMTs. Figure 17 shows an array of SiPMs and the equivalent circuit.

The dimensions of the microcells are of the order of 7 × 7 μm^2^ to 70 × 70 μm^2^. In such a system, many microcells (of the order of thousands) may be available in parallel connection with each other. The response of G-APDs is quite fast, of the order of 1 ns, and their photo-efficiency is satisfactory (35% at 550 nm) [178,179].

Application-specific integrated circuits (ASIC) that offer various functionalities are employed to read out the SiPMs output. Field programmable gate arrays (FPGA) are also used in some systems. SiPM sensors are characterized by their *photon detection efficiency* (PDE), which is defined as follows (Equation (36)):

(36) $$PDE\left(\lambda ,V\right)=QE(\lambda )G\varepsilon_{geiger}(V)$$

where *λ* is the wavelength, *V* is the voltage applied, QE is the quantum efficiency, *G* is the geometric efficiency (active over total sensor area), and ε_geiger_ the probability of a carrier created in the active mass to initiate a Geiger-mode discharge. Values of 15% to more than 60% have been reported [180].

A very critical property of SiPMs is the timing performance often expressed as the single-photon time resolution (SPTR), giving the efficiency to record the time of arrival of a detected single photon [180]. Values of some tens of ps, with a desired value of 10 ps, have been reported [125,180].

In analog SiPMs, the output signal, which is proportional to the total amount (energy) of incident light, is transferred to the readout and digitization stages. In the so-called *digital SiPMs* (dSiPM), these stages are integrated into the SiPM wafer [181] (e.g., SiPM technology is implemented in CMOS) [43]. Advantages of digital SiPMs are that they do not need read-out electronics such as ASICs, they show lower dark counts, and they improve timing performance [43].

As it has been referred to, SiPMs have a number of drawbacks, such as non-linearities in their response, cross-talk between cells, temperature dependence of their gain, dark counts (thermally generated), and gain dependency on the voltage applied [170]. However, significant progress, related to novel developments in the field as well as in overcoming drawbacks (PDE, noise, crosstalk, timing performance), has been reported, showing that SiPM is becoming the sensor of choice in PET scanners [180]. Currently, SiPM sensors are developed with CMOS technology [125,163,182].

Summarizing in terms of the development and properties of optical sensors, we could conclude that CMOS (and particularly APS-CMOS) technology is promising for many reasons, among which is the integration of electronic circuits in the same device (amplification, signal modulation, digitization, timing, etc.) and on-chip operation, size reduction, low power consumption, rapid image acquisition, allowing for random access to image data, low noise compared to CCDs, low production cost, a sufficiently established manufacturing process, etc. However, they present relatively high dark current. Another promising technology, particularly for nuclear medicine applications, is SiPM, which exists either in analog or in digital form. SiPMs have a small size high PDE, operate under low voltages (with respect to PMTs), exhibit very good timing resolution, have a high integration of readout and processing, is compatible with magnetic fields, and flexibility in sensor design, etc. SiPM may be implemented in CMOS technology [72,73,125,161,162,164,176].

## 5. Integrated Systems

A description of common full imaging detectors (i.e., DR, DF, CR, FFDM, CT, SPECT, PET, etc.) will be given in the following text. In the framework of LCSA, an integrated imaging system can be analyzed as follows [65,66,67,68,69,70,72,73,74,75,135,162,184]: Stage 0, radiation incidence (X-rays or gamma-rays), Stage 1, detection of incoming radiation (gain g_1_), Stage 2, conversion of radiation into optical photons or charge carriers (gain stage g_2_), Stage 3, transmission of optical photons to the converter output, Stage 4, spreading (blurring) during stage 3 (blurring stage T_4_(ν)), Stage 5, optical photon detection and photoelectron generation in optical sensors (not applicable in direct conversion systems) (gain stage g_5_), Stage 6, spreading (blurring) in the optical sensor element (photodiode, etc.), (blurring stage) T_6_(ν), Stage 7, aliasing effects in the photodiode array sampling, Stage 8, additive electronic noise.

The output signal (number of information carriers) of an imaging radiation detector may then be expressed as follows (Equation (37)):

(37) $$N_{T}=\bar{\Phi_{0}}\bar{g_{1}g_{2}g_{4}g_{5}}$$

The blurring effects are expressed through the MTF by the product (Equation (38)):

(38) $$MTF\left(u,v\right)=T_{4}(u,v)T_{6}(u,v)T_{7}(u,v)$$

For the noise power spectrum, it holds (Equation (39)):

(39) $$NPS\left(u,v\right)=\bar{\Phi_{0}g_{1}g_{2}g_{3}g_{5}}\left[1+\bar{g_{3}g_{5}}\left(\bar{g_{2}}+\varepsilon_{g_{2}}\right)T_{4}^{2}\left(u,v\right)T_{6}^{2}\left(u,v\right)\right]{T_{7}^{2}\left(u,v\right)}^{**}III(u,v)]+{NPS}_{add}$$

III(u,v)] is the sampling grid Fourier transform (see Equation (3)). The additive electronic noise *NPSadd* (or *σ*_1_^2^ + *σ*_2_^2^ + *σ*_3_^2^ + … in space domain) may include pixel noise, which includes photodiode dark current, TFT thermal noise, shot noise and 1/f noise, data-line thermal noise, noise from the power supply, preamplifier noise, and digitization noise of the ADC. This analysis is principally applied in X-ray imaging; however, it can be adapted to any imaging system. Details of the above formalism can be found in [69,70,72].

### 5.1. Digital Radiology (Radiography and Fluoroscopy)

Digital X-ray radiography and fluoroscopy systems are among the most technologically advanced and very widespread in the daily routine of hospitals and health care units. Radiography of the chest and other anatomical regions of the human body (including dental radiography) are the most common imaging examinations throughout the world. Research into the development of these systems, as well as radiotherapy portal imaging systems, is very active and ongoing [69,70,72,115,158,159,168,169,172,175,184,185,186,187,188,189,190,191,192,193,194,195,196,197,198,199,200,201,202,203,204,205,206,207,208]. Traditionally, X-ray radiography was performed by conventional radiographic cassettes consisting of a double-coated AgBr emulsion (film) sandwiched between two phosphor screens (CaWO_4_, Gd_2_O_2_S:Tb, LaOBr, etc.) (SF systems). The image is formed on the film, which is also a means of final display. In current digital X-ray imaging, image creation is divided into two main stages: (a) in the image formation process and (b) in the final display on a screen or on a hard copy. Digital processing techniques can be applied to improve or modify the final image. The X-ray tube voltage ranges from 50 to 150 kVp for general projection imaging and 20 to 40 kVp for FFDM. *Digital tomosynthesis* (DTS) is a technique used to acquire a limited number of projections over a relatively narrow range of angles to produce images with a partial separation of overlapping tissues after applying image reconstruction methods. DTS is used in mammography, in chest imaging, etc., and is based on similar detector technology as in ordinary digital radiography. Silicon strip photon counting detectors have also been proposed and used [158]. *Electronic portal imaging devices* (EPID) are another branch of medical imaging detectors integrated into radiotherapy accelerators. They are employed to acquire images of patients under treatment, using the megavoltage accelerator photon beam for geometric and dosimetric verification. Detectors of this kind are very similar to digital radiography flat panels, described in the next section. In most radiography and fluoroscopy systems, special devices aiming to absorb scattered radiation, referred to as *anti-scatter grids,* are placed between the patient and the detector. They consist of lead strips (to absorb scatter), separated by strips of a low-atomic-number-material easily penetrated by primary radiation. Anti-scatter grids may have parallel or converging (relative to the X-ray tube) strips. These devices improve image quality (contrast), however, at the cost of increasing the dose.

In diagnostic radiology, image formation can be modeled by Equation (40):

(40) $$i=\int_{E}\Psi_{Q}(E)exp(-\int_{0}^{Z_{0}}\mu_{t}(n_{i},\mu_{i},E,z)dz)\prod_{i}g_{i}\left(E\right)dE+i_{s}(SPR)$$

where *i* is the image signal, *Ψ_Q_* is the X-ray energy fluence of the collimated beam incident on the human body, and *μ_t_* is a function depending on the X-ray attenuation coefficient of the chemical elements in human tissues. *μ*_i_, *n_i_* is the number of atoms of the chemical element *i*, *g_i_* denotes the signal conversion stages (X-ray detection, conversion into light, conversion into electric charge, etc.), $i_{s}$ is the signal produced by scattered radiation, which is a function of the scatter to primary ratio (SPR). Integration is performed over the X-ray spectrum. The term in brackets expresses the presence and the corresponding attenuation of the human body.

Artificial intelligence and deep learning techniques have found extensive application in medical imaging in general and diagnostic radiology in particular. The related techniques started with chest X-ray radiography and mammography and have now expanded to such an extent that opinions are expressed that in a few years the medical specialty of the radiologist will be unnecessary [27,28,49].

#### 5.1.1. Active-Matrix Flat Panel Imagers—AMFPI or FPD

**Figure 18:**
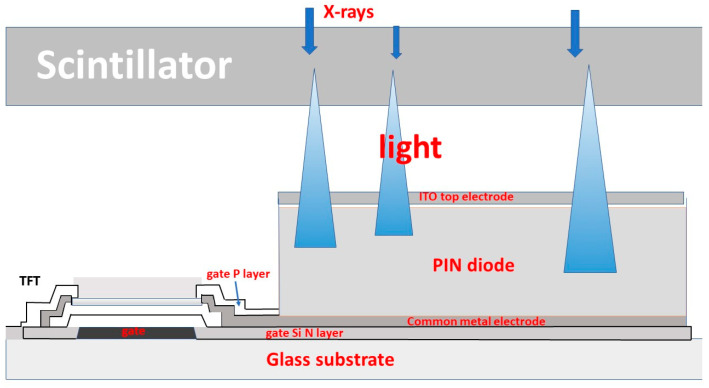
Detector element of an indirect conversion detector with CsI:Tl or Gd_2_O_2_S:Tb scintillator [184].

**Figure 19:**
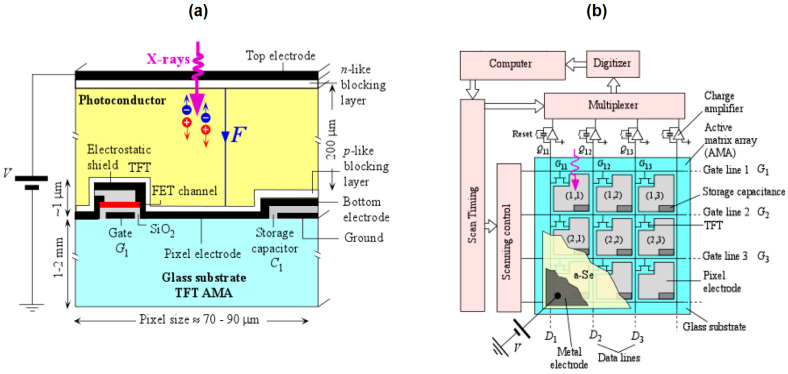
(**a**) detector element of a direct conversion system with an a-Se photoconductor and (**b**) corresponding active matrix [134].

**Figure 20:**
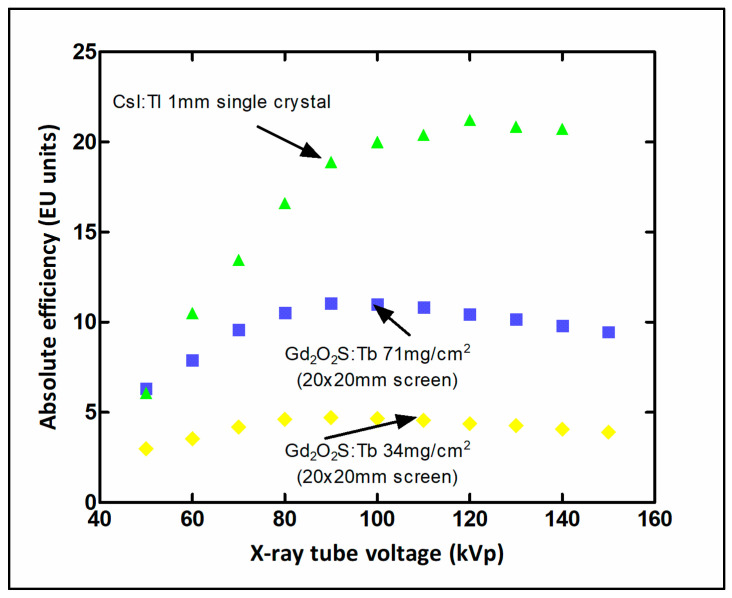
Variation of X-ray absolute luminescence efficiency (light energy flux over X-ray exposure rate), with X-ray tube voltage for phosphor-scintillator screens used in radiography and fluoroscopy (EU units: (μW/cm^2^)/mR)).

On top of an AMA (photodiodes and TFTs), there is either a 150–400 μm thick phosphor screen (CsI:Tl or Gd_2_O_2_S:Tb) or a coating of photoconductive material, e.g., an a-Se plate 200–1000 μm thick, depending on the application (Figure 18 and Figure 19) (i.e., thin coatings are used in FFDM, where lower X-ray tube voltages, 25–40 kVp, are employed). Coating thickness is often expressed as surface density (e.g., 30–150 mg/cm^2^). Detector area dimensions may be 35 × 43 cm^2^ (e.g., for chest radiography) or 18 × 24–24 × 30 cm^2^ for mammography. Such detectors used in DR, DF, and FFDM may contain large numbers of optical sensor elements (e.g., up to 4288 × 4288 photodiode arrays with pixel pitch between 50 and 200 μm or even 20 μm with CMOS technology [1,3,12,13,19,66,68,69,70,71,72,73,134,135,162,163,164,168,169,174,175,184,186,188,189]. Gd_2_O_2_S:Tb (often referred to as Gadox:Tb) is a dense material (7.34 g/cm^3^) with a high effective atomic number (61.1), very high ICE (i.e., fraction of absorbed X-ray energy converted into light, 0.15) emitting at 545 nm, well compatible with the spectral sensitivity of many optical sensors. Its decay time is 600 μs, which is not too slow for radiography. The surface of the phosphor material facing the X-ray beam is covered by a polyester reflective support containing TiO_2_. This increases sensitivity (speed) by reflecting light towards the optical sensor. However, this coating may reduce spatial resolution since it increases light spread. Dense materials show high sensitivity (X-ray absorption) and good spatial resolution since, for a given mass, a high-density coating is thinner, thus limiting light spread. CsI:Tl is of lower density (4.51 g/cm^3^), lower effective atomic number (54) and lower ICE (0.10) than Gd_2_O_2_S:Tb; however, its needle-like crystal structure restricts lateral light spread and improves spatial resolution (needle diameter: ~1–30 μm). In addition, its crystal transparency improves light output. Hence, thick coatings may be used to improve sensitivity. Fiber optic plates (FOP) (~1–3 mm thick) are often used between scintillator and optical sensor with fiber diameter 1–3 μm (FOS/fiber optics scintillator detectors). The emission spectrum of CsI:Tl is centered at 550 nm, which is also compatible with many optical sensors. Its decay time is 900 ns, and it is slightly hygroscopic. Both Gd_2_O_2_S:Tb and CsI:Tl show high light yield, around 60–65,000 ph/MeV, being the most efficient of scintillators. Figure 20 shows experimental data on luminesce emission efficiency of these scintillators. In many newly developed systems, CMOS optical sensors are incorporated in conjunction with fiber optics or optical lenses. In the direct conversion systems, the photodiodes act as storage capacitors.

#### 5.1.2. CCD-Based X-ray Detectors

In these systems, a Gd_2_O_2_S:Tb, Gd_2_O_2_S:Eu, or CsI:Tl phosphor screen is deposited on a CCD array via FOP, optical lenses, or even directly [6,12,19,172]. Since CCDs are small (40–60 mm^2^), a demagnifying FOP is used with fibers of gradually decreasing diameter. The light-collection efficiency of the optical system has low values and constitutes an amplification stage that degrades the overall efficiency. These detectors have been used mainly in digital mammography systems (either in large surface systems or in linear—one-dimensional detectors).

#### 5.1.3. Computed Radiography (CR)

**Figure 21:**
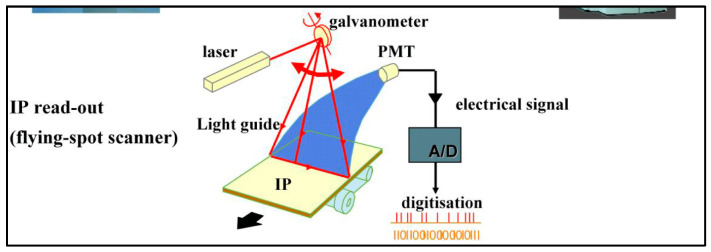
Computed radiography system and imaging process [115].

These systems use storage phosphors to absorb X-rays and store their energy. To start the emission, the phosphor must be excited by a laser beam (photoexcitation effect). The phosphor (BaFBr:Eu^2+^ or CsBr:Eu^2+^) is used in the form of a flat granular screen (with grain size 1–15 μm and 100–300 μm thickness) inside a cassette (image plate-IP), similar to the conventional intensifying screens. Needle-like structured phosphors (RbBr:Tl^+^) have also been used. After being irradiated, the phosphor is placed in a special image read-out device. Inside this device, a thin laser beam scans (in raster scanning) the surface of the phosphor, and then every point in this surface emits light. The light is collected by a suitable fiber optic system and directed to a photomultiplier or some other kind of optical sensor (Figure 21). The latter produces an electrical signal proportional to the intensity of the light. This is followed by digitization via an analog-to-digital converter. The laser beam spot (50–100 μm) serves as the aperture, which is related to the sampling frequency and spatial resolution. Read-out of the plate lasts less than 60 s and read-out of the pixel is 2 μs. To stimulate the next pixel, the emission of the previous one should have dropped to very low levels. This poses restrictions for the decay time of the phosphor. The IP is erased before the next radiograph, since only 50% of the stored energy is emitted after irradiation by the laser beam. Computed radiography systems are very successful applications in diagnostic radiology. They can be used with conventional radiography systems, replacing conventional radiographic (film-screen) cassettes. They are widely used in clinical practice but are characterized by a not-so-fast workflow due to the processing requirements of the cassette. They also require higher doses than DR, mainly due to the fluorescent materials they use [31,115].

#### 5.1.4. Silicon Strip Detector

This type of detector consists of silicon strips (e.g., n-silicon wafers with p-doped strips forming a pin diode, depleted by a voltage of 150 V) arranged in *edge on geometry* to the X-ray beam (with the strips parallel to the incident beam). The thickness of the wafers is several hundreds of μm with a 50 μm pitch. Pre-breast collimators as well as post-collimators are available. The detector modules have an effective thickness of some mm. Silicon strips are coupled to amplifying and pulse shaping electronics [158,159,197,198]. Such detectors have also been considered for spectral CT applications (see Section 5.2) [209].

#### 5.1.5. Avalanche Imagers HARP and SHARP Detectors

Detectors of this kind are based on the avalanche effect occurring in a-Se photoconductors (discussed in Section 3.2). SHARP (Scintillator HARP) configurations use a scintillator layer (i.e., CsI:Tl) on top of an a-Se (in avalanche process). The optical photons of the scintillator are absorbed by the a-Se layer, and then they are captured in an a-Si TFT AMFPI matrix [132].

#### 5.1.6. Image Intensifier-Based Detectors

Image intensifiers are traditional X-ray detectors consisting of an evacuated glass tube incorporating an input phosphor screen (CsI:Na), 300–500 μm thick, coupled to a photocathode emitting photoelectrons. Electron lenses and an anode (at 25–35 kV) focus and accelerate electrons, which fall on an output phosphor screen (ZnSCdS:Ag) that convert electron energy into light photons. The latter are captured by a CCD camera. These systems have been traditionally used in conventional fluoroscopy and in some older digital radiography systems. Accelerated electrons produce very bright images at the output screen, thus thereby creating conditions for reducing X-ray flux and dose.

*The imaging performance of digital radiology systems* is estimated by determining the MTF, NPS, and DQE following the IEC 62220-1-2 and IEC 62220-1-1:2015 standards [207,210,211]. To avoid aliasing, in digital imaging, the *pre-sampling MTF* is determined by using high sampling frequency. A tungsten edge test device (PTW L659136), slightly inclined with respect to the pixel matrix, is used to obtain the image of an edge (edge spread function-ESF) and then after differentiation of the LSF. MTF is determined after Fourier transformation [115]. NPS can be determined [115] by the Fourier transform (FT) of a large area flat field image *I*(*x_i_*,*y_i_*). This flat field image is separated into M regions of interest (ROIs) of 256 × 256 pixels half-overlapping with each other. Then, the NPS is expressed as (Equation (41)):

(41) $$NPS\left(u,v\right)=\frac{\Delta \chi \Delta y}{MN_{x}N_{y}}\sum_{m=1}^{M}{\left|\sum_{i=1}^{N_{x}}\sum_{j=1}^{N_{y}}\left(I\left(x_{i},y_{j}\right)-\bar{I}\left(x_{i},y_{j}\right))exp(-2\pi i(u_{n}x_{i}+v_{k}y_{j})\right)\right|}^{2}$$

where Δ*x* and Δ*y* are the pixel pitches in the x- and y-directions, *Nx* and *Ny* are dimensions of a region of interest (ROI) in the image, and *I*(*xi*,*yi*) is the flat field image for subROI i as a function of x and y. The normalized version, NPS, is often used for comparison purposes. The normalized noise power spectrum (NNPS) is defined by dividing NPS with the square of the mean pixel value ${\bar{\mu }}^{2}$. DQE can then be experimentally estimated according to IEC standard following Equation (42) [207,210,211]:

(42) $$DQE\left(v\right)=\frac{{MTF}^{2}(v)}{K_{a}qNNPS\left(v\right)}$$

where *q* is the number of photons per unit kerma (μ*Gy*) per mm^2^, determined by dividing the number of photons per mm^2^ with the corresponding air-kerma ($K_{a}$) value (μ*Gy*).

### 5.2. X-ray Computed Tomography

**Figure 22:**
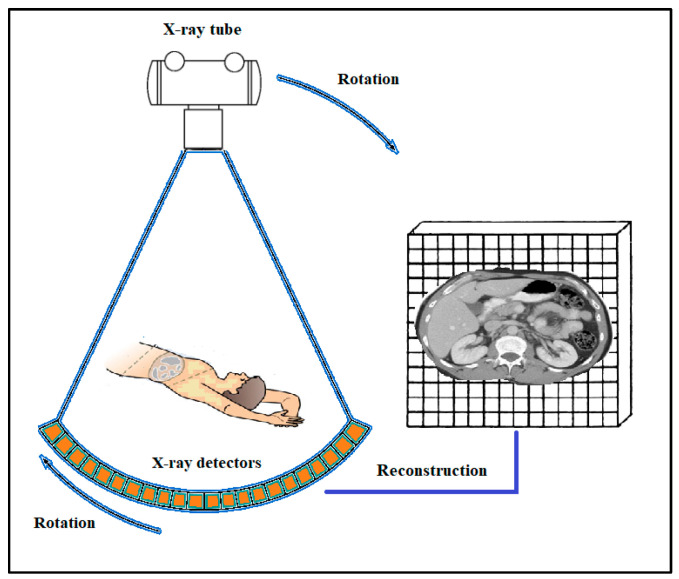
Detector arrays are arranged in an arc-shaped configuration [230].

The invention of tomographic image reconstruction revolutionized medical imaging and was awarded a Nobel prize (in Physiology and Medicine, 1979). Research developments are particularly impressive in both detector design and in reconstruction algorithms [20,21,23,24,25,26,29,173,209,212,213,214,215,216,217,218,219,220,221,222,223,224,225,226,227]. In X-ray CT, the image of an unknown object is reconstructed from its multiple projections on detector arrays. This can be achieved by an arc-shaped detector array rotating around the object (Figure 22). Radiation has a spectral distribution, produced by X-ray tubes operated at 80–150 kVp (mean energy after human body transmission: 50–70 keV) [228]. Images can be obtained in a few seconds, with gantry rotations in 0.25 s. This is due to increased speed of gantry rotation, multidetector rows, and fast reconstruction algorithms (with deep learning-based methods among them) [23,24,25,26,27,28,29,38,48,50,51]. Patient dose burden is an issue of major concern in CT examinations. This has been addressed by introducing *low-dose CT* based on changing imaging parameters, iterative reconstruction, and AI methods. The latter have been employed to enhance image quality and reduce noise. Deep learning reconstruction (DLR) has been introduced to create images of high quality with a low dose faster than other techniques.

**Figure 23:**
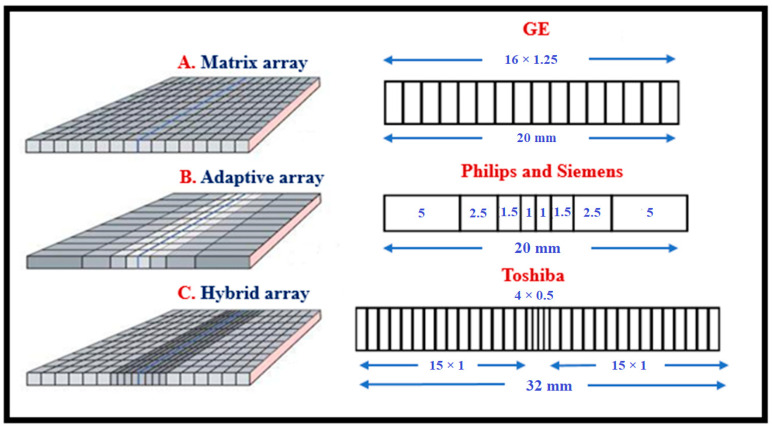
Array patterns of CT detectors. In a real system, the flat surfaces are curved, as shown in Figure 22.

Two-dimensional detector arrays, termed *multirow detector computed tomography* (MDCT), are employed in current CT machines. The number of imaged slices may be 32,64,128 etc., up to 320 or even 640. In general, detector arrays are classified into three main patterns (Figure 23) [229]: 1. Isotropic detector arrays or matrix arrays. In these arrangements, the detectors are all of equal dimensions (e.g., 0.5 mm, 1 mm, or 1.25 mm). 2. In the so-called adaptive detector arrays with different dimensions (e.g., along the direction of movement of the table it will be: 5 mm, 2.5 mm, 1.5 mm, 1 mm, 1 mm, 1.5 mm, 2.5 mm, 5 mm). 3. Hybrid arrays. Slices may be of different thickness depending on the number of detector elements irradiated. The thickness of the beam, and therefore the number of rows irradiated, is determined by collimators at the output of the X-ray tube.

Adaptive detector arrays have better geometric efficiency, i.e., they receive a higher fraction of emitted radiation. This is due to the fewer number of septa between detectors. On the contrary, the process of determining the slices is more complex. Adaptive layouts are present in many 16-slice systems. An example is given of a system comprising 24 rows of detectors, of which the central 16 (from the fifth to the twentieth row) have detectors approximately 1.5 mm wide. The 8 extremes (first through fourth and twenty first through twenty fourth) have 3 mm range detector elements. The corresponding slice thicknesses (at the center of rotation) are 0.75 mm and 1.5 mm. Adaptive arrangements are also used in 32-slice systems. Most 64-slice systems use 64-row isotropic arrays, as well as the 256-slice system with an equal number of detector rows. Lately, systems with isotropic arrays and detector elements with 0.25 × 0.25 mm^2^ entrance surface dimensions, arranged in 160 rows, have been commercially available, named ultra-high resolution (UHRCT) [231,232]. Spatial resolutions of the order of 0.14 mm and 4.1 lp/mm at MTF = 10% have been reported.

Systems based on active matrix flat panel large surface area detectors have also been developed (see previous section) [233]. These systems are characterized as volume-computed tomography scanners (VCT) (*flat panel volume CT*). In such a system, the whole arrangement includes 2048 × 1536 elements, each of which has dimensions of 194 μm^2^. Using such a detector, 1536 slices can be acquired in one rotation. A key feature of these systems is the high spatial resolution, since in projective-radiographic imaging this resolution is of the order of 150–200 μm. Data acquisition time can reach up to 100 frames per second. The problems presented are related to: (a) increased scattered radiation since a large area of the body is irradiated; (b) reduced QDE due to the thin fluorescent screen (compared to the thickness of the scintillators in conventional detectors); (c) the time response (decay time) of CsI, which is slower than that of conventional detectors (see UFC detectors: ultra-fast ceramics); (d) the dynamic range, which is relatively limited compared to ordinary CT scanners. Another similar instrumentation is *cone beam computed tomography* (CBCT), which is a method based on two-dimensional area detectors, using 3D divergent pyramid (“cone”) shaped beams to image head and neck, principally applied in dentistry. CBCT methods are also used in radiation therapy, either using a megavoltage X-ray beam (MV-CBCT) or a kilovoltage beam (kV-CBCT). CBCT allows precise target localization and dose calculation. Detectors and X-ray tubes (in kV-CBCT) are mounted on the linear accelerator unit. Recently, cone beam breast computed tomography (CBBCT) systems have also begun to be developed [234,235,236,237]. Electron beam tomography (ΕΒΤ) is another method in which X-rays are produced by a beam of electrons circularly scanning a half-ring of tungsten, which surrounds the patient. Images are formed at high speed, and thus cardiac imaging becomes possible. Finally, the *dual-source CT* systems, having two X-ray tubes and two detector rows working simultaneously for very fast scanning, should be referred.

**Figure 24:**
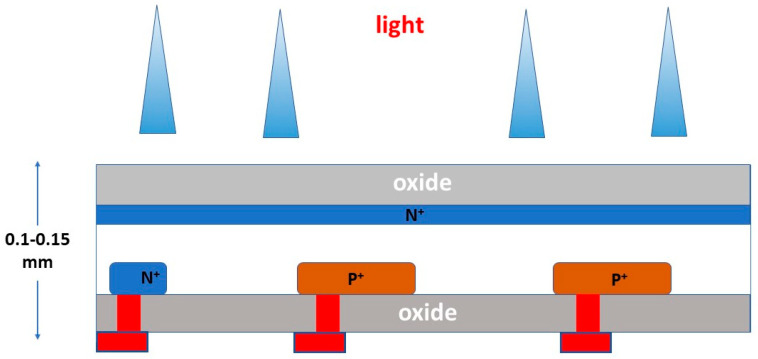
Photodiode (working in photovoltaic mode) [212].

CsI:Tl and CdWO_4_ scintillators have been traditionally used in CT. Most modern CT detector configurations use ceramic scintillators such as (Gd_2_O_2_S: Pr, Ce, Fe, and Cd_2_O_2_S:Pr), garnet materials (Y,Tb,Lu)_5_Al_5_O_12_:Ce, or scintillators with europium (Eu) activator, Y_2_O_3_ (Eu), and Gd_2_O_2_ (Eu) in contact with crystalline silicon photodiodes (c-Si) or even amorphous silicon (a-Si) (Figure 24).

Ceramic scintillators (Gd_2_O_2_S, etc.) are prepared by melting (sintering) the fluorescent material at high temperatures and pressures. The material is initially in powder form. The response (or decay) time to irradiation is short (3μs), and the name “ultra-fast ceramics” (UFC) has been established. High transparency to their own light and low levels of afterglow are several useful properties of these materials. Other promising materials are (Lu,Gd,Y,Tb)_3_ (Ga,Al)_5_O_12_ [212]. Packaging of the scintillator should, include a reflective material to avoid light escaping from the side surfaces except the surface in contact with the photodiode. Some general characteristics of scintillator detectors for CT are: 1. high but uniform sensitivity and light yield of all detector elements; 2. low quantum noise; 3. light transparency; 4. fast decay time; 5. spectral matching with photodiodes; 6. ease of correcting response instabilities with conventional electronic techniques; 7. compact packaging.

Photodiodes (PD) used in CT detectors, in addition to good spectral matching with scintillators, should also have linearity within 0.1%, high values of shunt resistance (used to protect the semiconductor) to reduce noise, and low crosstalk between detectors. Following PDs there is the data acquisition system (DAS) including low-noise preamplifiers-integrators, after each PD, to integrate the current from PDs and convert it into voltage, the ADCs (16 bit) and multiplexers. Electronic noise must be significantly lower than quantum noise, while a broad dynamic range is required. The integration time must follow the rotation time requirements.

**Spectral CT.** One interesting area of research and innovation in medical imaging is *spectral CT.* Spectral CT techniques aim to investigate tissue physical properties depending on the radiation energy within the X-ray spectrum. Different X-ray energy spectra may be used to this aim. Dual-energy CT is one of such methods. More specifically, the methods of spectral CT based on the use of specialized detectors are the following:

*1*. *Dual layer detectors*
*2*. *Photon counting detectors (PCCT)*

**Figure 25:**
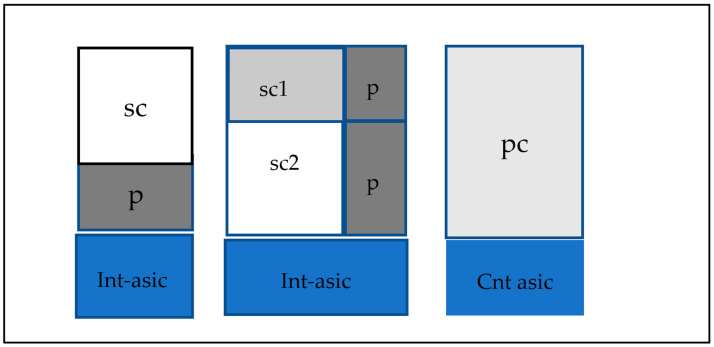
CT detector element configuration: Left, monolithic scintillator (sc) and dual-layer scintillators (sc1: low Z, sc2: high Z) coupled to a photodiode (p) and direct conversion detector (pc). Integrating asic (int-asic), counting asic (cnt-asic) [212].

In the dual-layer method, the scintillator consists of two materials with different X-ray attenuation coefficients (i.e., differing in effective atomic number and density) and different thicknesses (Figure 25). On top of the detector configuration is placed the thin (e.g., 1 mm) scintillator material with a low attenuation coefficient to absorb preferably low energy photons (e.g., ZnSe, YAlO, etc.). Below is a thick (e.g., 2 mm) high absorption coefficient scintillator (e.g., Gd_2_O_2_S). The optical sensors (i.e., silicon photodiodes) are thin and placed laterally on a vertical side of the two-scintillator array. This configuration maintains detector pitch and geometric efficiency similar to conventional detector arrays. In this method, both X-ray energy data are acquired simultaneously and at identical angles.

Photon counting detectors are becoming more and more popular during the last two decades in CT systems. These detectors are based on direct conversion materials, mostly CdTe, CZT, Si, GaAs, etc. They have smaller detector elements (pixels), and X-ray photons are analyzed according to their energy, thus allowing for dual and multi-energy imaging with a single detector. They can work with a lower dose and achieve higher CNR. Additionally, the lower size of detector elements can ameliorate spatial resolution. Due to the X-ray spectrum acquisition technique, the effective energy of the incoming photons used for image creation can be reduced, thus increasing contrast. They provide the possibility of processing individually single radiation photons and applying energy weighting techniques (i.e., use of suitable energy weighting factors to characterize photons). They can suppress low-frequency noise and dark current as well as the so-called Swank noise (see Equation (12)). Energy thresholds can be applied to exclude unwanted parts of the X-ray or γ-ray spectrum (e.g., originating from Compton scattering). They have linear response, better spatial resolution and contrast, and improve dose efficiency [156,157,238].

To overcome patient dose burden problems, novel techniques have been adopted, i.e., the *sparse view CT*, with decreased number of projections and *interior view CT* where only a small part of the anatomic region is irradiated.

The imaging performance of CT systems can be evaluated by measuring MTF, NPS, and DQE similar to those used in diagnostic radiology, i.e., the MTF can be estimated by taking the Fourier transform of PSF or ESF [225,239], following Equation (43):

(43) $$MTF\left(v\right)=\left|\int_{0}^{\infty }LSF(x)exp[-2\pi ivx]\right|dx/\int_{0}^{\infty }LSF(x)dx$$

However, some particular image quality parameters for CT are the following [212]:

1. Spatial resolution (full width at half maximum of PSF) is given by Equation (44)

(44) $$R_{0}=\frac{1}{M}{\left[d^{2}+{\left(M-1\right)}^{2}s^{2}\right]}^{1/2}$$

where *d* is the dimension of detector elements, *s* is the focal spot of the X-ray tube, and *M* is the magnification. As it can be seen, PSF degrades (i.e., R_0_ increases) with increasing detector element dimension.

2. The detection efficiency (DE) is given by Equation (45):

(45) $$\eta_{D}=\eta_{G}DQE\sim \eta_{G}\frac{{MTF}^{2}(f)}{NPS(f)}$$

where *η_G_* is the geometric efficiency, expressing the fraction of emitted X-ray photons arriving at the detector. An index commonly used to compare CT systems in terms of radiation dose output is the *computed tomography dose index* (CTDI)_W_, defined as the weighted average of dose across a slice.

### 5.3. Single Photon Emission Computed Tomography-γ Camera

SPECT (and γ-camera) is by far the most widespread system of nuclear medicine. Low cost and ease of delivery of radiopharmaceuticals are among the reasons for this. Common SPECT functional examinations refer to the heart, brain, bones, etc.

Image formation in nuclear medicine can be modeled by Equation (46):

(46) $$i=\frac{A_{0}exp(-\lambda t)}{4\pi z^{2}}exp(\int_{0}^{z_{0}}\mu_{t}\left(z\right)dz)ACF(z)\prod_{i}g_{i}$$

where *A*_0_ is the initial activity of the radiopharmaceutical injected in the patient’s body, *λ* is the decay constant of the radionuclide, *μ_t_* is the γ-ray attenuation coefficient of the human body, which should be as low as possible, *ACF* is the attenuation correction factor required in order to compensate for the attenuation of radiation falling on the detector, and *g_i_* are the conversion stages of the detector system. ACF is necessary since in nuclear medicine the radiation emitted from a particular anatomical region must be depicted before penetrating human tissues (γ-ray photons must escape the body without interaction). The factor *4πz*^2^ accounts for the inverse square low of uncollimated emitted radiation attenuation. SPECT systems are the most common type of nuclear medicine imaging system [151,240,241,242,243,244,245,246,247,248,249,250,251,252,253]. It is employed in monitoring diseases (cardiovascular system, brain functions, central nervous system, cancer, etc.) and in functional studies at molecular level. However, the objective image quality of SPECT is clearly degraded by many factors intrinsic to the imaging process. AI methods have been systematically employed to overcome image quality issues as well as to improve image processing, analysis, and reconstruction techniques [30,37,54,55,56]. The energy of the γ-ray photons may be 140 keV (from ^99m^Tc), 364 keV (from ^131^I), etc.

**Figure 26:**
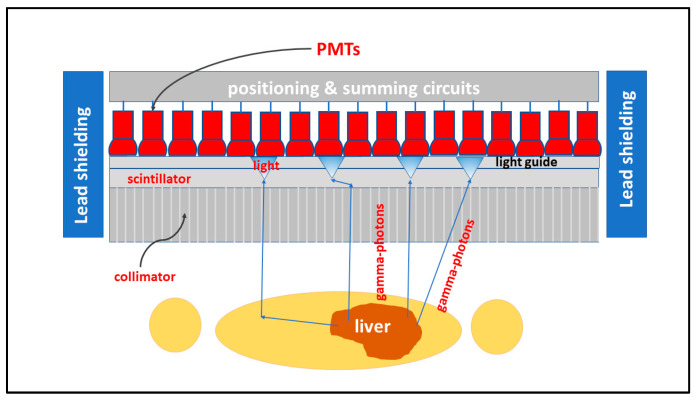
γ-camera detector, including collimator, scintillator, light guide, photomultipliers, position-determining unit, and shielding.

SPECT systems are based on the γ-camera detector configuration. A complete γ-camera system consists of one, two, or three detector heads with the support and movement mechanisms (gantry). Each detector head includes a collimator (see below), a scintillator, a light guide to facilitate light collection by the optical sensors, the sensors (i.e., photomultipliers, photodiodes, etc.), the electronics part, including preamplifiers, linear amplifiers, pulse height-analyzing devices (to perform γ-ray spectrometry), and some additional electronics. Most nuclear medicine imagers in use today are based on the principle of Anger’s camera detector, consisting of a large-dimension single-crystal NaI(Tl) or CsI:Tl scintillator coupled to an array of 30–100 photomultipliers. In addition to Anger’s type, which uses a single crystal gamma camera, there are other types, e.g., with many microcrystals (multi crystal camera), etc. The basic structure of the detector head of an imaging system is shown in Figure 26. First the collimator can be seen, which in most cases is of the multi-parallel hole type. In some cases of small organs, a pinhole-type collimator is used (see next). *Collimators* are flat-plate-shaped components that have multiple parallel, converging or diverging holes through which photons pass (Figure 26). The holes may be round, square, triangular, or, in most cases, hexagonal. They may also have a single hole (pin hole). They are made from high-atomic number materials (lead, tungsten) and determine the size of the anatomical region to be imaged as well as the direction of the primary γ-photons to be detected. Depending on the γ-ray energy and on the spatial resolution required, collimators of various dimensions (thickness, hole diameter, etc.) are used, which are divided into low energy high resolution (LEHR), high energy high resolution (HEHR), medium energy (ME), etc. The collimator restricts the rays from the source so that each point in the image corresponds to a unique point in the source. Through the collimator anatomical regions are projected on the scintillator surface. Behind the collimator is the scintillator crystal (NaI:Tl and, in some cases, CsI:Tl). Usually it is of rectangular shape, covered by a reflective material (TiO_2_) to reduce front and lateral light losses, and by a thin aluminum sheet (less than 1 mm) to protect it from moisture. In the rear crystal surface there is an optical coupling (window or light guide) with refractive index between that of the photocathode and the scintillator to minimize light losses and to allow light photons to reach the PMTs. The dimensions of the crystal are over 50 cm × 40 cm (e.g., 57.5 cm × 45 cm), usually 6.7–12.5 mm thick. Regarding the PMTs, sensors of round, hexagonal, and square entrance surfaces have been used. These developments aim to reduce dead zones between tubes uncovered by the scintillator. The number of PMTs for a given detector area is a matter of cost. Large entrance surface sensors reduce the number of PMTs and the total system cost and simplifies readout electronics. However, light sharing problems arise that affect image accuracy. The use of smaller tubes at the edges of the camera has been favored as a solution to such problems. The PMTs current is converted to voltage by suitable amplifiers, then digitized by ADCs and fed to FPGAs. In any case, full digital cameras increase the readout and processing complexity (e.g., a large number of high-speed ASCs, etc.) as well as the total detector cost.

A crucial aim of the electronics part of a nuclear medicine system is (a) the determination of the position from which the photon γ originates and (b) the spectroscopic determination of the γ-ray energy in order to control its originality. Pulse height analyzers as well as amplifying and pulse shaping electronics (preamplifiers, amplifiers) are necessary components in the measuring chain of nuclear medicine detectors. In principle, the pulses from the photomultipliers are combined in such a way that in the end only two pulses are obtained, characterized as pulse X and pulse Y. The intensities of these two pulses are proportional to the x and y coordinates of the point of γ-photon absorption. Traditionally, X and Y pulses are calculated by the use of a resistor matrix. Each PMT is coupled to four resistors having values depending on their distance from the center of the array, thus depicting coordinates of points of γ-ray interactions. In modern detectors, X and Y pulses are determined by digital computations (Equation (47)).

(47) $$X=\frac{\sum_{i}x_{i}Q_{i}}{\sum_{i}Q_{i}},\;Y=\frac{\sum_{i}y_{i}Q_{i}}{\sum_{i}Q_{i}}$$

where *Q_i_* is the output signal of the *i_th_* PMT, *x_i_*,*y_i_* denotes the position of the PMTs [14,16]. The denominator in these formulas provides the sum of pulses from all PMTs, which is related to the energy of the incident γ-ray photon. According to these formulas, the position determination is independent from the energy of the incoming γ-ray photon. The pulse corresponding to the denominator passes through the pulse height analyzer to spectroscopically determine whether the photon originates from the radioactive source within the patient’s body. The aforementioned method is referred to as the Anger logic or center of gravity (COG) multiplexing algorithm. However, COG images suffer from spatial resolution and linearity problems. One method that has been developed in combination with the use of rectangular PMTs in all digital cameras is the digital correlated signal enhancement (CSE) method designed to minimize image quality problems [254,255].

A dedicated brain SPECT scanner has been developed and configured in the form of two arcs, approximating a ring detector. Detector modules are arranged in three-arc shaped rows incorporating NaI:Tl crystals and a number of converging collimators covering three detector elements each. Cardiac SPECT is also an important tool in detecting coronary artery (CAD) disease [147,246].

Systems based on CZT semiconductor detectors have been recently developed and entered the market. CZT detectors are direct conversion systems in which the stages of conversion of γ-rays into light and light into electronic signal are omitted, i.e., there are fewer stages of signal degradation and noise creation. These systems have relatively compact size consisting of a large number of detector elements, e.g., 1024 (32 × 32) with dimensions 2.46 × 2.46 × 5 mm^3^. The energy resolution of CZT detectors (<6%) is clearly better than that of scintillators (11–13% at 140 keV).

**Figure 27:**
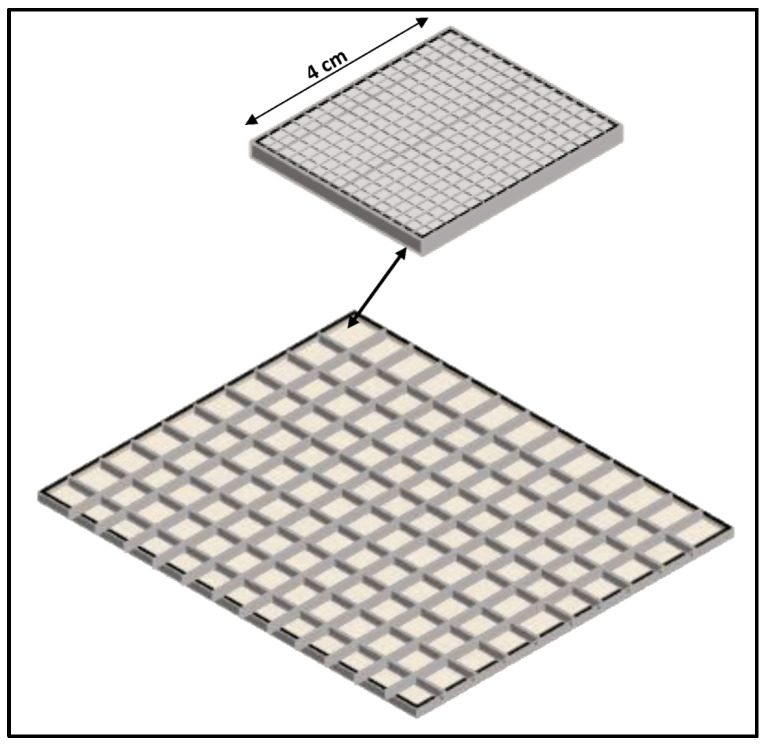
CZT detector arrays for SPECT [147].

In initial configurations, the detectors consisted of arrays of elementary CZT cells (Figure 27) [152,153]. The thickness of the detector material was initially up to 2 mm and later was 4 mm (with increased detection efficiency and sensitivity).

The overall dimensions are significantly smaller than in a conventional γ-camera. In the existing systems to date, they can reach more than 25 × 35 cm^2^. Some of the existing layouts include: 1. Detector with 40 × 32 CdTe:Cl detector elements (pixels), each element size 4 × 4 mm^2^, with Pt contacts and total dimensions of 16 × 16 cm^2^. 2. Detector with 64 individual modules with dimensions of 25 × 25 × 5 mm^3^ each. Each unit consists of a CZT detector consisting of 8 × 8 elements (pixels) measuring 3 × 3 mm^2^. Pulse shaping circuits are also included in each such unit. The total active area of this detector is 21.6 × 21.6 cm^2^. 3. Detector composed of some monolithic CZT or CdTe sub-units coupled to multiplexers. The disadvantage of the measurement process is that there is increased recombination or trapping of carriers (electron-holes). Therefore, a number of the generated charges are not collected by the electrodes. It has been proven that a technique to reduce the problem consists in the use of elementary cells whose surface dimensions are smaller than their thickness. This technique is based on the so-called “near field effect” [152]. One parameter used to evaluate detectors for this characteristic is the charge collection efficiency (see Section 3.3).

**Figure 28:**
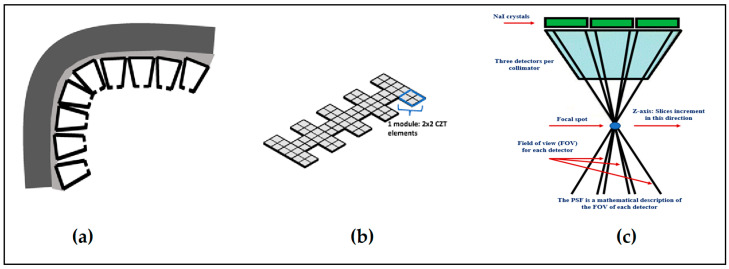
(**a**) SPECT with CZT crystals arranged in an arc or in an L shape configuration; (**b**) schematic representation of detector configuration; (**c**) detector array profile with pin-hole collimators [147].

In a more recent configuration, there are two rotating heads incorporating a 10 × 13 matrix of detectors, each one including a 16 × 16 matrix of detector elements of side 2.46 mm (Figure 27) [147]. Another cardiac-dedicated system consists of nine 4 cm × 16 cm arrays of CZT detectors with tungsten parallel hole collimators, rotating around their central axis. The detectors are arranged in an arc shape to focus the heart. The collimators have a larger aperture than length to increase sensitivity, however with lower spatial resolution. In another configuration, dedicated to cardiac imaging, the CZT crystals are arranged in an arc or in an L-shaped configuration (Figure 28). 19 detector modules, each consisting of 2 × 2 detector elements with 16 × 16 pixels each, along three lines perpendicular to the patient axis. The detector array follows a 3-1-3 scheme, i.e., the central line comprises 9 modules, while the other two lines include 5 modules in an 1-0-1 arrangement (alternating detector-vacancy, no detector-detector, etc.). 27 pinhole collimators are placed in front of each module (including vacancies), i.e., in front of 4 detector elements. The system’s central spatial resolution is 5.8 mm. The sensor spatial resolution is 2.46 mm. The energy resolution is 5.4% at 140 keV [57,147]. Such systems achieve significantly higher sensitivity, according to manufacturers. What mainly distinguishes this configuration from traditional Anger-logic cameras is its tomographic count sensitivity, which is up to four times higher [147]. In such systems, the tracer dose is significantly reduced. Thus, follow-up studies can be followed.

The system of collimators is designed so that each collimator covers a block of four CZT crystals. Their geometry is that of a pinhole, with an opening of 5.1 mm, focusing on a small volume in order to have the quality field of view (QFOV) within which the patient’s heart is positioned. Due to the pinhole shape, sensitivity increases with decreasing distance, and detection of photons from tissues and organs not included in the QFOV is reduced. The energy resolution, already very good in CZT crystals, is further ameliorated by the presence of oblique angles of incidence due to the pinhole geometry (Figure 28).

In general terms, the use of CZT-based detectors contribute to energy and spatial resolution improvement; system sensitivity (electrons per absorbed γ-ray photon) increases, thus requiring less activity and shorter acquisition time. In addition, with these detectors, a large fraction of the scattering part of the γ-ray spectrum may be avoided, thus improving the signal-to-noise ratio. Finally, such cameras are very compact [146].

Image quality measures in SPECT are similar to those used for CT in the spatial frequency domain (i.e., MTF, etc.). However, of particular interest in nuclear medicine are the following definitions (considering a circular lesion in a healthy tissue):

1. Spatial resolution R_0_ (full width at half maximum of PSF) is given by Equation (48):

(48) $$R_{0}={\left[R_{c}^{2}+R_{i}^{2}+R_{s}^{2}\right]}^{1/2},\;\;R_{c}=\frac{d}{l}[l+b+c]$$

where *R_C_* is the contribution of the collimator, *R_i_* is the contribution of the rest of the system (detector, electronics), and *R_S_* is due to scattering, *l* is the thickness of the collimator, *d* is the diameter of the holes, *b* is the distance from the patient body, and *c* is the distance between the collimator and crystal. The second relation on the right holds only for the parallel hole collimator.

2. Contrast is given by Equation (49):

(49) $$C=\frac{\left(R_{L}+R_{B}\right)-\left(R_{0}+R_{B}\right)}{R_{0}+R_{B}}$$

*R_L_* is the count rate originating from a circular lesion, *R_B_* is the count rate due to background radiation, and *R*_0_ is the count rate originating from health tissue surrounding the lesion.

3. Contrast to noise ratio is given by Equation (50):

(50) $$CNR=\frac{C}{C_{N}}={Cd}_{L}{\left(R_{0}t\right)}^{1/2},\;\;C_{N}=\frac{\sigma_{Ν}}{Ν}$$

*C_N_* is the noise contrast, *d_L_* is the diameter of the circular lesion, and *N* is the mean number of γ-ray photons.

### 5.4. Positron Emission Tomography

**Figure 29:**
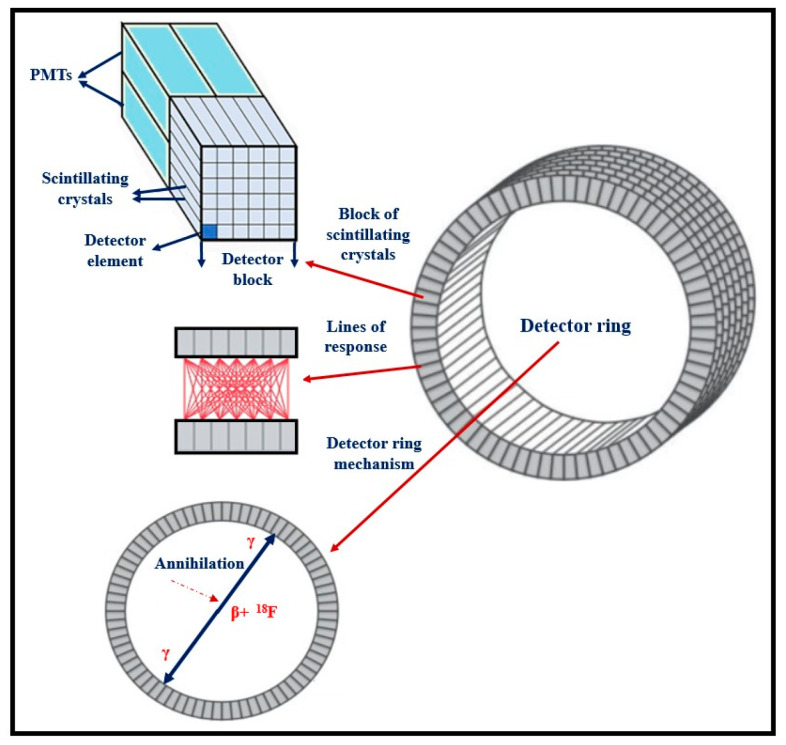
Left: PET system showing detector elements and system configuration; right: detectors with and without septa.

Positron emission tomography is a nuclear medicine imaging method employing positron (β^+^) emitters and providing biochemical information in vivo at the sub-pico-molecular level [43,125,170,171,180,181,182,256,257,258,259,260,261,262,263,264,265,266,267,268,269,270,271,272,273,274,275,276,277,278,279,280,281,282]. Cancer, neurologic disorders, and cardiovascular diseases are among PET focus areas. The positron emitters (^15^O, ^11^C, ^18^FDG/fluorodeoxyglucose) show a similar behavior to biological molecules, which reinforces the functional nature of PET exams (FDG due to glucose-G). In addition to routine use in hospital conditions, it is also widely used in preclinical research in biological and pharmaceutical sciences. Research in PET detector materials and instrumentation design is significant and rapidly evolving. Particular uses of artificial intelligence in PET include attenuation and scatter correction, determination of photon position and depth of interaction (see below), time of flight (see below), image reconstruction, etc. [30,48,52,54,55,56,161]. PET detectors are mostly based on scintillator-optical sensor (PMTs, APDs, SiPMs) schemes working with photon counting and photon coincidence techniques. For each positron, two 511 keV photons are created traveling in opposite directions. These photons are detected by two opposing radiation detectors (Figure 29), connected by a coincidence circuit, which rejects photons not originating from the same annihilation event. Scintillators for such applications must fulfill the following requirements: (i) fast response (decay time) to match the requirements of coincidence-based measurements (the term coincidence time resolution—CTR, is often used); (ii) high quantum detection efficiency for the 511 keV annihilation photons. For such photons, the probability of photoelectric absorption (depending on Z_eff_^3^) is lower than that of the Compton effect for most scintillator crystals. Additionally, Compton is the most probable interaction of these photons in water (simulating the human body), and their mean free path length is approximately 10 cm [249]. The connection of the crystal to the optical sensor follows: (a) either the “light sharing” design (one crystal provides light to multiple sensors or one sensor collects light from multiple small crystals) or (b) the “one-to-one” design (one crystal—one sensor). Optical sensors can be arranged in several ways: (a) sensors on the back side of the crystal; (b) sensors on the front side of the crystal; (c) sensors on the side surface of the crystal; (d) sensors on both the front and back sides of the crystals [259].

Most PET machines today are combined with CT systems in one unit to provide both functional and morphological information. PET has evolved significantly during the last decades in terms of spatial resolution and sensitivity. The latter is important in order to reduce Poisson noise. The dominant design of a modern PET system is a circular detector consisting of detector rings that surround the patient (Figure 29). The ring consists of several distinct detector modules. Each building module consists of individual detector blocks, which in turn contain a planar array of several individual scintillation crystals. The individual crystals are elongated (e.g., 20, 25, or even 30 mm thick) with transverse dimensions of 4 × 4 mm^2^, 3.95 × 5.3 mm^2^ or lower, etc. In the literature, the term “crystal segmentation” is used. For example, a PET system can be composed of tens (50–60) blocks that contain several modules (e.g., 2 × 3, 8 × 8, 13 × 13) with a few tens (30–40) crystals in each module. The total number of crystals will be in the range of 12–24,000. Annihilation photons are incident and absorbed by the crystals, and their energy is converted into light photons. The light is then guided, through optical coupling, having an intermediate index of refraction (in some systems via fiber optics), to an array of photomultipliers (e.g., four in each building block). The photomultipliers are position-sensitive photomultipliers (PS-PMT). APDs and SiPM are also used. PS-PMTs have the ability to determine the coordinates of the specific scintillator crystal that absorbed the maximum energy of the annihilation photon. Optical photons produced in the crystals are emitted isotropically in all directions. However, crystal segmentation (an array of many thin individual rectangular rod-like crystals) prevents light spreading in a large area of the detector array and the escape of photons from one crystal to another. Consequently, the photons are mainly directed towards the photomultiplier located in the rear side of the crystals. The position of high-energy photon incidence is determined by multiplexing techniques, e.g., by the relative ratio of the signals at the output of the photomultipliers. The coordinates (x,y) of this position are calculated through Equations (51) and (52):

(51) $$x=\frac{{(x}_{11}+x_{12})-{(x}_{21}+x_{22})}{x_{11}+x_{12}+x_{21}+x_{22}}$$

(52) $$y=\frac{{(x}_{12}+x_{22})-{(x}_{11}+x_{21})}{x_{11}+x_{12}+x_{21}+x_{22}}$$

where *x_ij_* the signals of the four photomultipliers (considered as algebraic matrix elements).

APDs have also been investigated for use in PET detectors, particularly in combined PET-MRI systems. APDs have lower gain than PMTs, but they can be produced in very small dimensions, either incorporated in compact monolithic arrays or in small devices. They do not exhibit any magnetic susceptibility up to more than 9 T, and thus they can be used with MRI coils. SiPM sensors seem to be the most promising technology for currently developing TOF-PET instrumentation. Signals from analog sensors are digitized and fed to readout electronics (FPGA, ASIC). In digital sensors (e.g., dSiPMs), readout electronics are incorporated in the sensor chip.

Traditionally, BGO and CsF scintillators have been used in PET detectors. In recent years, new materials have been used, such as LSO:Ce (abbreviation of Lu_2_(SiO_4_)O:Ce), (Lu,Y)_2_SiO_5_:Ce (LYSO:Ce), GSO:Ce (Gd_2_(SiO_4_)O:Ce), YAP:Ce (abbreviation of YAlO_3_:Ce), LuAP:Ce (LuAlO_3_:Ce), YSO (Y_2_(SiO_4_)O:Ce), etc. In Table 4, the quantum detection efficiencies of some common scintillators are presented. These materials present very short decay times, e.g., 40 ns for LSO and 28 ns for YAP. The short times are mainly due to the presence of the activator Ce^3+^ (cerium). LSO has a high density (7.4 g/cm^3^), a high effective atomic number (Zeff = 66), is compact, not fragile, non-hygroscopic, and has a high conversion efficiency of absorbed radiation into light (25–30,000 optical photons/MeV, almost 60–75% of that of NaI). It has an energy resolution of 9.1% at 511 keV. However, it presents an intrinsic radioactive emission (300 Bq/cm^3^) due to the natural presence of a small proportion of radioactive Lu-176 together with stable Lu. The optical emission spectrum shows an emission maximum at 420 nm. GSO has a density of 6.7 g/cm^3^, an effective atomic number of Zeff = 59, and a decay time of 60 ns. YAP: Ce has a satisfactory density (5.37 g/cm^3^) but consists of elements with low atomic numbers (39, 13, 8). The conversion efficiency of the absorbed radiation into light is sufficient (50% of that of NaI). The maximum of the emission spectrum is at 370 nm. The presence of the Ce^+3^ activator causes the emission of blue light, which shows high spectral compatibility with photocathodes of photomultipliers.

Crystals activated with two ion activators, i.e., LYSO:Ce, Ca (where Ca is divalent), have been proposed, showing higher emission efficiency and a short decay time [125]. However, despite all this, BGO is still interesting due to its low production cost and high QDE.

**Figure 30:**
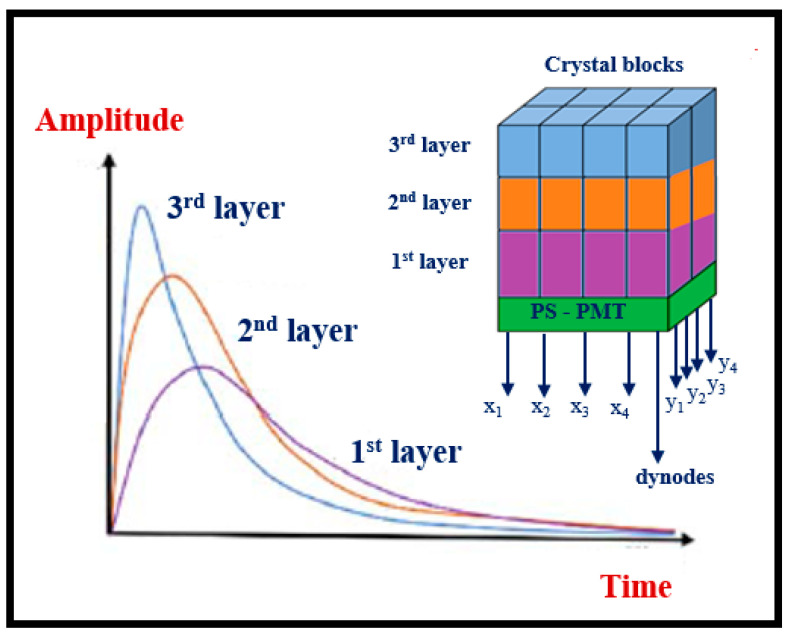
The phoswich detector module is comprised of three layers of individual crystals, each with a different output pulse shape.

*Phoswich detector* designs with *depth of interaction* (DOI) capabilities have been developed to avoid resolution degradation by parallax effects [181]. This is achieved by using two or more crystal layers of different materials with different decay times and different pulse shapes (e.g., BGO, LSO, LYSO, GSO, etc.), coupled with some optical grease between them. Interaction events occurring in different crystal layers are distinguished by methods based on decay time and pulse differences (Figure 30).

**Figure 31:**
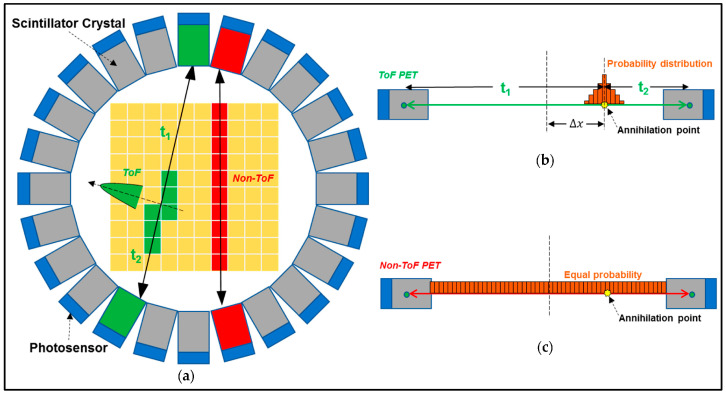
(**a**) PET detector ring, illustrating the time of flight (ToF) technique and detected annihilated gamma photons with ToF (green) and without (red), (**b**) The probability distribution of the annihilation position along the line of response (LoR), (**c**) Illustration of the equal probability of the annihilation position, along the line of response in non-time of flight scanners [161].

*Time of flight PET* (TOF-PET) is a significant development based on techniques estimating the position of annihilation along the line of response (LOR), i.e., the difference between the times of annihilation photon interactions with the detectors (Figure 31). The accuracy *Δx* of position estimation is determined by the accuracy Δt of time difference of photons incidence on the detectors (Equation (53)) [170].

(53) Δx=cΔt/2

Detector properties for such application may be deduced from the following relation of figure of merit (Equation (54)):

(54) $$FOM=\varepsilon_{i}^{2}\varepsilon_{g}\frac{D}{\Delta t}\frac{1}{cost}$$

where *D* is the diameter of the object to be imaged, ε_i_ is the intrinsic detection efficiency, ε_g_ is the geometric efficiency, and *cost* is the cost of the detector [125]. Scintillators must exhibit very fast rise and decay times. Materials such as LYSO:Ce, BaF_2_,LaBr_3_:Ce, and CsF, as well as the co-doped LYSO:Ce, Ca, and LFS-3, LGSO:Ce, LuI_3_:Ce, and GAGG:Ce (Gd_3_Al_2_Ga_3_O_12_:Ce), have been used or proposed for use [32,125,171].

Regarding the dimensions of the crystals, it should be noted that thick crystals assure high detection efficiency, while narrow ones are required for precise position localization. Hence long and narrow scintillation elements (of low aspect ratio, i.e., crystal width over crystal length) have to be used in detector arrays [43]. A representative crystal element size is 3 × 3 × 20 mm^3^. Crystal dimensions of 2.45 × 2.45 × 15 mm^3^, 2.35 × 2.35 × 15.2 mm^3^, etc. have been reported. However, thick crystals may have lower light transmission efficiency and show light losses. The latter can be avoided (to some extent) by improving internal reflection properties (crystal face polishing, suitable etching) and/or by using crystals of lower length (10 mm instead of 20 mm). One solution that improves DOI and TOF performance of crystals is the dual-side readout, i.e., coupling optical sensors on both crystal’s faces or coupling a one-dimensional array of optical sensors on one of the 3 mm × 20 mm crystal sides. In addition, to avoid light photons cross talking between crystal elements, optical reflecting layers are placed in between. Another issue is the complexity and cost of procedures for cutting, polishing, and assembling crystals [43,125].

**Figure 32:**
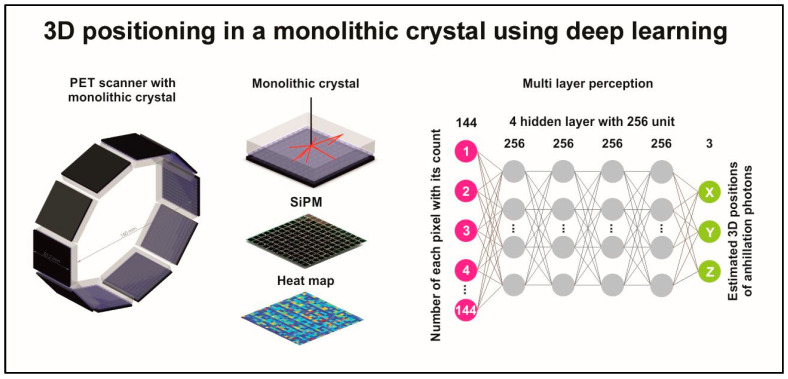
Monolithic crystal detectors for PET, read by SiPM and using machine learning to estimate annihilation position [264].

Another approach is the use of a monolithic crystal (e.g., LYSO:Ce) block (slab) of relatively large size, coupled to individual or position-sensitive optical sensors [151,249,262,264] (Figure 32). Machine learning methods are used to estimate the position of interactions. This is determined using a multilayer perceptron (MLP) neural network, implemented as a deep neural network (DNN), which ameliorates significantly spatial resolution (clearly lower than 2 mm) [264]. Analytical and statistical methods have also been used. Detector sensitivity is maximized by this design since there are no gaps between detector elements, while cost is reduced.

**Figure 33:**
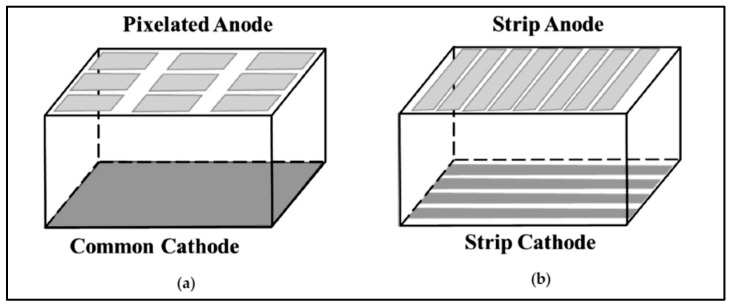
Pixelated and cross-strip detector configuration of CZT PET detectors (**a**) pixelated; (**b**) cross-strip pattern [161].

CZT -based PET detectors have also been developed, exhibiting very good energy and spatial resolutions. CZT has a relatively good effective atomic number (49.1 in the case of Cd_0.9_Z_0.1_Te); however, at 511 keV, the fraction of photoelectric absorption, as compared to the Compton effect, is low (0.18 and 0.82, respectively). The energy required to create an electron-hole pair is 4.64 eV [161]. Electrode configurations in such detectors follow two designs: the pixelated anode and the cross-strip design (Figure 33). In the case of pixelated detector elements, only the anode is pixelated, consisting of an array of rectangular electrodes (e.g., 8 × 8 elements with pixel pitch 2.2 mm). The cathode is not pixelated, and the detector body has dimensions of 19.4 × 19.4 × 6 mm^3^ [161]. The whole system comprised two such detectors. Other systems that have been investigated were based on 22 × 22 × 15 mm^3^, 40 × 40 × 15 mm^3^, pixelated units. Low energy resolution values (3.7–1.5%) can be achieved in these systems [161].

In the cross-strip configuration, anode and cathode strips are deposited on each side of the crystal element (e.g., 16 anodes and 5 cathodes in a crystal volume of 20 × 16 × 0.9 mm^3^). One such development uses anode and cathode cross strips to sample the interaction position along two dimensions. The cathode-to-anode signal ratio is used to determine the position in the third dimension. The cross-strip configuration requires relatively fewer electronic readout channels (than a fully pixelated anode). In the CZT detectors, detector pixilation is implemented electronically. Spatial resolution of 1 mm can be achieved, and energy resolution lower than 2.5% has been reported. These CZT detectors are then stacked in an edge-on configuration to provide high detection efficiency.

Drawbacks of CZT are the relatively high attenuation coefficient for Compton scattering at 511 keV and the low temporal resolution. A high mass attenuation coefficient leads to a significant fraction of multiple interaction photon events (MIPEs). It is crucial to recover true coincidences to gain high-contrast images. In a high spatial resolution PET system based on CZT, a cross-strip electrode configuration can be utilized to reduce the number of readout channels. However, this configuration leads to more ambiguity in paring anode-cathode in intra-detector scatters. Another drawback is timing jitter that makes it difficult to use CZT in TOF-PET applications [161].

The full CZT detector system incorporates: (a) a charge-sensitive amplifier to integrate signal (current), giving the total charge and converting it into voltage, and, in addition, to provide signal matching in the detector—electronics chain; (b) a shaping amplifier, which, apart from amplification, minimizes noise.

In summary, it could be said that the ongoing research in PET technology, to a large extent, aims at improving the entire detection chain and all kinds of related parameters in order to reduce the CRT, i.e., the co-incidence resolving time expressing the temporal accuracy in determining the photons arrival at detectors input, while simultaneously maintaining a high level of detection efficiency and spatial resolution. CRT values in the range of 214–325 ps are reported in the literature with the ultimate goal of reaching 100 ps and even 10 ps [125].

In PET system evaluation, parameters such as the scatter fraction (SF) and noise equivalent count rate (NEC or NECR) are often used, defined as follows (Equation (55)):

(55) $$SF=\frac{N_{S}}{N_{S}+N_{0}}$$

(56) $$NECR=\frac{{{(N}_{0})}^{2}}{N_{0}{+N}_{S}+kN_{T}}$$

where *k* is a constant with values between 0 and 2, depending on the used correction techniques for random measurements. *N*_0_ is the counting rate from true coincidence, N_T_ is the counting rate corresponding to the random coincidences, and *N_S_* is the counting rate from scatter coincidences. The spatial resolution, i.e., the FWHM of PSF (often referred to as CPSF: coincidence PSF), has been estimated by Equation (57):

(57) $$R_{FWHM}=1.25{\left[{\left(\frac{d}{2}\right)}^{2}+{\left(0.0022D\right)}^{2}+R^{2}+b^{2}\right]}^{1/2}$$

*d*: crystal pitch, *D*: scanner diameter, *R*: effective positron range, *b*: a factor empirically determined from BGO, block detectors that ranges from 0 to 2.2 depending on detector design [151,253,283]. Conventional PET systems exhibit a resolution of 4–5 mm; however, values lower than 2 mm have been reported [161].

**Total body PET**: These systems have a long axial field of view (e.g., 195 cm) to increase sensitivity and cover a large part of the human body [270,281,282]. These systems show high resolution, fast imaging, and allow for total body dynamic scanning, providing real-time images. One such system consists of 564,480 LYSO:Ce crystals arranged in 13,340 blocks with dimensions of 2.76 × 2.76 × 19.1 mm^3^ coupled to 53,760 SiPM optical sensors. The spatial resolution is 3 mm. A CT system with 80 detector rows is incorporated in the machine. Another system has a 140 cm axial dimension and 3.86 × 3.86 × 19 mm^3^ LYSO:Ce crystals coupled to digital SiPMs [281].

### 5.5. Magnetic Resonance Imaging

Although this study focuses mainly on ionizing radiation systems, a reference will also be made to magnetic resonance imaging, due to the increasing importance of hybrid systems. Magnetic resonance imaging is a morphological imaging method based on the nuclear magnetic resonance (NMR) of hydrogen nuclei [14,18,147]. Methods of functional imaging (fMRI) have also been developed within this framework. In MRI, relatively strong magnetic fields (1.5–3 T or higher), produced by superconductive or permanent magnets, are used to align the magnetic moments of protons within the human body. Protons found in a magnetic field occupy two quantum energy levels, one corresponding to parallel alignment (low energy level) and the other to antiparallel alignment (high energy level) of the magnetic moments. The difference in the population of protons in the two levels results in the creation of an intrinsic magnetic field (magnetization) within the human body. These levels correspond to an energy difference of hω_0_, where ω_0_ is the resonant frequency of protons. After excitation by radiofrequency magnetic pulses with ω_0_ frequency, the protons absorb energy, and soon after they de-excite, emitting a RF signal of the same frequency. In the MRI terminology, this process is called relaxation. The latter is divided into longitudinal and transverse relaxation, both showing exponential behavior, with corresponding time constants (T_1_, T_2_ relaxation times). To obtain a spatial differentiation of RF signals in accordance with the point of emission within the human body, field gradients are applied, which spatially modify the frequency of signals as functions of coordinates. A series (sequence) of RF pulses is normally applied to enhance image contrast. The latter depends on hydrogen concentration in various tissues, relaxation times, and flow and diffusion phenomena within the human body.

The principal parts of MRI systems are. 1. The main magnet producing the static magnetic field (from less than 1T up to more than 10 T). Superconductive magnets are mostly made from an Nb-Ti alloy. They consist of a number of rings (e.g., 5–7) containing a thin (2 mm) Nb-Ti wire surrounded by copper. Other materials are also used, characterized as high-temperature superconductors-HTS (i.e., MgB_2_), often with a different shape, such as pancake coils, with a cross-section of 0.2 × 4 µm^2^. 2. Gradient coils, producing gradient fields for spatial determination of the imaged area. 2. Shim coils, for the smoothing of the local inhomogeneities of the static magnetic field. 3. RF coils. for the production of radio frequency (RF) pulses and also for the detection of induced magnetic resonance signals (free induction decay—FID). They are distinguished into transmitter coils and receiver coils, although signal emission and reception can be performed with the same coil. Birdcage shape is most often employed for RF coils, although TEM (transverse electromagnetic) structures have been recently introduced. There are also separate surface coils that are placed in contact with the patient’s body for better signal reception. Correspondingly, the coils that completely enclose the anatomical area under consideration are characterized as volume coils. The RF circuitry used to drive the RF coils consists of the transmitter and the receiver circuit, often referred to as the transceiver. In modern systems, most individual transceiver units are digital; e.g., many functions, such as signal modulation and filtering, are implemented through logic circuits using FPGA elements. Fiber optics as well as wireless signal transmission are being currently adopted in MRI RF sensors.

### 5.6. Ultrasonic Imaging

A very short description of ultrasonic imaging (USI) may be of value at this point, since ultrasound is employed in some hybrid imaging systems technologies. This kind of imaging is based on a pulse-echo technique, i.e., ultrasound waves fall on and penetrate through the human body, within which various physical effects, such as reflection and refraction, scattering (and back-scattering in particular), Doppler, as well as absorption, occur [14,18,147]. A small fraction of the ultrasound wave energy is back-reflected and/or back-scattered and finally captured by an ultrasonic sensor. The latter is usually a piezoelectric transducer that also serves as a source of wave generation. Piezoelectric transducers convert electrical energy into pressure waves and vice versa. They are made of piezoelectric ceramics (lead zirconate titanate) or combinations of polymers with piezoelectric ceramics. Transducers are configured in arrays of crystal elements, either linear, curved, or two-dimensional crystal arrays. Ultrasonic beams are formed, focused, and guided through wave interference effects on the emissions from the elementary crystals. Other technologies, such as microelectromechanical elements, e.g., cMUT and pMUT, i.e., capacitive and piezoelectric micromachined transducers, and optical ultrasound detection, have emerged. The latter are based on the bending of a thin membrane coupled to a piezoelectric film [147].

### 5.7. Hybrid Systems

Nuclear (SPECT and PET) imaging provides mainly physiological information and almost ignores anatomical details, thus making it difficult to spatially localize the radioactive distributions, and accordingly the image diagnostic value is lower than expected. For this reason, PET (and sometimes SPECT) is combined with anatomical imaging systems, such as CT and MRI, in one unit. Functional (PET, SPECT) and anatomical (CT, MRI) data can be recorded in one image. CT and MRI provide good resolution anatomical images with soft tissue contrast and thus can significantly contribute to the spatial and anatomical delineation of PET signals. Additionally, CT and MRI data can contribute to PET image reconstruction, and in addition, the magnetic field can have a positive effect on the image quality since, due to Laplace-Lorentz forces, it can limit positron range and improve spatial resolution.

SPECT/CT design is based on the combination of two systems similar to those of the standalone CT and SPECT instruments or on the combination of a 40 × 30 cm^2^ flat-panel low-dose cone-beam CT (CBCT) with traditional γ-cameras. The two detectors are mounted on the same gantry [249].

In PET-CT instrumentation [271,272,273], 64–128-row CT detectors are now incorporated in current systems, and LYSO:Ce SiPM PET detectors have been employed. CT serves as a means of attenuation correction for PET measurements and, in addition, provides morphological images of high-quality, improving lesion detection and other diagnostic tasks. Modern PET-CT systems have dose-modulating systems to reduce dose. In the case of PET-MRI [274,275,276,277,278,279,280], particular problems arise as follows: (a) The presence of PET causes inhomogeneities in the static magnetic field with implications for MRI image quality. This is due to magnetic susceptibility differences between ferromagnetic materials of electronics and shields as well as in the scintillator materials. (b) MRI field gradients induce eddy currents (foucault) in the PET components, which in turn distort the field and affect the linearity of the gradients. (c) Emission of electromagnetic signals from the electronic components of PET, which interfere with MRI coils with negative effects on the image quality. (d) MRI magnetic fields affect electron trajectories in optical sensors, especially in photomultipliers, i.e., Laplace-Lorentz forces can affect electron trajectory (bending). (e) There is also an effect on scintillator light production efficiency. This effect depends on the chemical composition of the materials and is partly due to the different radius of curvature of the electrons resulting from incident photon interactions, e.g., the Compton electrons. (f) Interference with PET electronics. Also present are radio frequency interferences from the MRI transmitter coil, which are often responsible for reducing the count rates of the PET detectors. In addition to these, the radio frequencies of the MRI also cause vibrations and heating.

**Figure 34:**
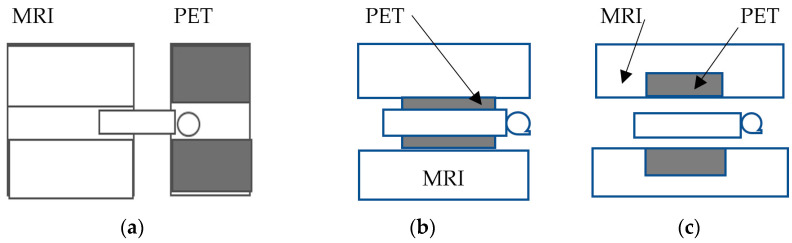
The three predominant PET-MRI hybrid system architectures. (**a**) tandem, sequence arrangement; (**b**) implant arrangement; (**c**) full integration arrangement.

The most common approaches to the design of PET-MRI systems are as follows (Figure 34): 1. One system behind the other (tandem or sequential arrangement), with a physical separation. This requires minimal modifications to the existing instrumentation, and the two exams take place consecutively, both spatially and temporally. 2. Insertion—adaptation of PET in the opening of the magnet (insert configuration). In this way, the two exams are simultaneously performed in the same place and probably at the same time. This configuration, however, requires a complete redesign of the PET system. 3. Complete and permanent integration of the PET system inside the magnet from the construction of the hybrid system (full integration layout).

For SPECT-MRI, one system (INSERT project) uses stationary rings of 10 × 5 cm^2^ detector modules, consisting of 8 mm thick monolithic CsI:Tl crystals coupled to SiPM sensors. A 38-channel ASIC board is employed for signal readout. This configuration inserts in an MRI system. 20 such detectors are arranged in a ring around the patient. RF coils are placed in between an aperture of 33 cm [250,251,252,253]. Other SPECT-MRI systems are based on CZT detectors and ASICs inserted in an MRI machine [252].

Hybrid *X-ray/MRI* systems have also been developed for interventional guidance [284,285]. These systems consist of an active matrix flat panel digital fluoroscopy detector and stationary anode X-ray tubes. The X-ray imaging system is positioned between two donut-shaped magnetic poles of MRI. Older systems within this technology used two separated units with an image intensifier and rotating anode tube, which are more sensitive to magnetic field effects.

Similar concepts of combining two or three imaging units have been developed by including *ultrasonic imaging* under various configurations [286,287,288]. PET-CT systems combined with ultrafast ultrasonic imaging (PET-CT-UUI) have been developed to yield simultaneously molecular, hemodynamical, biomechanical, electrophysiological, and structural information [286]. USI has also been combined with MRI, using a long cable and an aluminum foil shield to the USI transducer to minimize susceptibility artifacts [288].

*Medipix* is a series (Medipix 1, 2, 3, 4) of hybrid pixel detector readout ASICs, designed for particle tracking imaging and photon counting employed in various applications from particle physics to X-ray imaging and radiation therapy. They are based on a semiconductor n-diffusion layer (Si, GaAs, CdTe) over a p-type semiconductor. After interaction with radiation, the charge created is captured by pixel electrodes and then, through bump bonds, is guided to a CMOS electronic chip with single pixel readout cells [289,290].

### 5.8. Radiation Dose Burden from Imaging Modalities

The overarching goal of medical imaging systems is to obtain diagnostically reliable images and measurements while minimizing the radiation dose burden to the patient. For this reason, a brief reference is made to dose levels in the various branches of medical imaging. Average effective doses in usual radiographic examinations may vary in the range of 0.01 to 10 mSv, depending on the particular examination. Computed tomographic examinations have relatively higher average effective doses, varying approximately between 2 and 20 or even 34 mSv in some cases. In interventional radiology procedures, the range is from 5–70 mSv. In most nuclear medicine procedures, the average effective dose varies between 0.3 and 20 mSv. For comparison purposes, we refer to the fact that the average annual effective dose from background radiation is about 3 mSv [291,292].

### 5.9. Monte Carlo Methods

Monte Carlo techniques are very popular simulation methods of research applied in various areas of medical imaging and particularly in detector design and evaluation. Advantages of these techniques include the improved description of radiation transport as well as the simulation of physical parameters that are difficult or even impossible to determine by experimental measurements. Over the last decades, several general-purpose Monte Carlo radiation transport packages have been employed in medical physics [293,294]. EGS, PENELOPE, GEANT4, MCNP, PENELOPE, etc. are among the mostly used Monte Carlo codes. There are also Monte Carlo codes (e.g., MANTIS, LIGHTAWE) specifically designed to model and assess detective systems for medical imaging purposes [295,296].

## 6. Summary and Conclusions

This article provides an overview of the radiation detectors and sensors used in medical imaging systems. These detectors are based on three different types: (i) scintillators with optical sensors; (ii) photoconductors; and (iii) semiconductors. The last two types are characterized as direct conversion detectors and, according to the theory of linear cascade systems, are characterized by fewer signal conversion stages and therefore fewer points of signal loss and noise creation. Technological developments are accelerating with corresponding effects on the quality and on the workflow of results. Diagnostic radiology systems produce images of significantly higher quality; however, those in nuclear medicine investigate functional information even at the molecular level. Hybrid systems combining morphological and functional information are of increasing significance. Medical imaging systems must operate under conditions of low patient irradiation, i.e., minimizing input photon flux. This amplified the quantum noise and reduced the signal-to-noise ratio. In these conditions, the best way to evaluate the detectors is through the DQE, which is a key indicator of the quality of such a system.

## Figures and Tables

**Table 1:** Presents a summary of the most common imaging systems. M: morphological imaging, F: functional imaging, EI: energy integrating, PC: photon counting, D: direct conversion, I: indirect conversion, Phys Body: physical effects in human body, Phys Det: physical effects in detector, PE: photoelectric, C: compton, PP: pair production, XRD: X-ray diffraction, NMR: nuclear magnetic resonance, EMI: electromagnetic induction, R: reflection, Ab: absorption, BS: back scattering, Dpl: doppler effect, PZ: piezoelectric effect, +: indicates the category to which the system belongs.

	M	F	EI	PC	D	I	Phys Body	Phys Det
Screen-film radiography (SF) intensifying screens	+		+			+	PE	PE
Digital radiography (DR)	+		+		+	+	PE	PE
Digital mammography–(FFDM)	+		+	+	+	+	PE	PE
Computed radiography (CR)	+		+			+	PE	PE
Computed tomography (X-ray CT)	+		+	+		+	PE	PE
Digital tomosynthesis (DTS)	+		+	+	+	+	PE	PE
Dental	+		+			+		
Single photon emission tomography (SPECT)		+		+	+	+	none	PE
(Positron emission tomography (PET)		+		+		+	none	PE
Portal imaging (EPID)	+		+			+	C, PP	C, PP
Phase contrast imaging (PCI)	+		+		+		XRD	PE
Magnetic resonance imaging (MRI)	+	+ (fMRI)			+		NMR	EMI
Ultrasonic Imaging (USI)	+				+		R, Ab, BS, Dpl	PZ

**Table 2:** Properties of some luminescent materials used in medical imaging. ρ: density, Z: atomic number, K-edge: energy of maximum photoelectric absorption/only energies of the heavier elements are shown, E_g_: forbidden energy band gap (approximate values), LY: light yield. Data from [97,108,111], bandgap data: [110].

Scintillator/ Phosphor	Ρ (g/cm^3^)	ρΖ_eff_^4^	K-Edge (keV)	E_g_ (eV)	LY (Photons/MeV)	Wavelength	Decay Time (μs)	Hygroscopic	Afterglow
CaWO_4_	6.1	89	4.03/69.5/	5.2	20,000	420		No	No
Gd_2_O_2_S:Tb	7.3	103	52.2	>5 (5.3)	60,000	545	10^6^	No	No
CsI:Na	4.5	38	35.98/33.61	6.4	40,000	420	630 ns	Yes	No
CsI:Tl	4.51	38		6.4	54,000–66,000	550	1000	Yes	Yes
CdWO_4_	7.9	134	26.71/69.5		28,000	495	5 × 10^3^	No	Slight
Gd_2_O_2_S:Pr, Ce, F	7.3	103	50.2		35,000	510	4 × 10^3^	No	Slight
Gd_2_O_2_S:Pr (UFC)	7.3	103	50.2		50,000	510	3 × 10^3^	No	Slight
NaI:Tl	3.67	24.5	33.61	5.9	38,000	415	230 ns	Yes	No
Bi_4_Ge_3_O_12_(BGO)	7.1	227	90.5/11.1	5	9000	480	300 ns	No	No
Lu_2_SiO_5_:Ce(LSO)	7.4	143	63.3/1.8	6	26,000	420	40 ns	No	No
Gd_2_SiO_5_:Ce(GSO)	6.7	84	50.2		8000	440	60 ns	No	No
YAlO_3_:Ce(YAP)	5,5	7	17.03/	8	21,000	350	30 ns	No	No
LaCl_3_:Ce	3.86	23.2	38.9	6	46,000–50,000	330	24 (60%)	Yes	No
LaBr_3_:Ce	5.03	25.6	38.9	5.5	61,000–70,000	358	16	Yes	No
GAGG:Ce	6.6	54.4	10.36/52.2		42,000–57,000	520		No	

**Table 4:** QDE values at 511 keV for various crystal scintillators.

			Scintillator Thicknesses (cm)				
Scintillator Material	Density (g/cm^3^)	Attenuation Coefficients @ 511 keV	1	1.5	2	2.5	3
			QDE Values				
CdZnTe	5.76	0.090	0.403	0.539	0.644	0.725	0.787
Lu_2_SiO_5_:Ce	7.4	0.117	0.580	0.728	0.824	0.886	0.926
Bi_4_Ge_3_O_12_	7.1	0.135	0.617	0.763	0.853	0.909	0.944
Labr_3_:Ce	5.03	0.089	0.359	0.487	0.589	0.671	0.737
(Lu,Y)_2_SiO_5_:Ce (Lutetium 50%, Yttrium 50%)	7.1	0.089	0.470	0.614	0.719	0.795	0.851
(Lu,Gd)_2_SiO_5_:Ce (Lutetium 50%, Gadolinium 50%)	7.0	0.111	0.541	0.689	0.790	0.858	0.904
CeBr_3_	5.1	0.089	0.366	0.496	0.598	0.680	0.746
Gd₃Al₂Ga₃O₁₂:Ce	6.63	0.098	0.477	0.622	0.726	0.802	0.857
BaF_2_	4.83	0.094	0.365	0.493	0.596	0.678	0.743

## Data Availability

The original contributions presented in the study are included in the article; further inquiries can be directed to the corresponding author.
